# Solvability of the Non-Linearly Viscous Polymer Solutions Motion Model

**DOI:** 10.3390/polym14061264

**Published:** 2022-03-21

**Authors:** Andrey Zvyagin

**Affiliations:** 1 Department of Algebra and Mathematical Methods of Fluid Dynamics, Voronezh State University, Universitetskaya Pl. 1, 394018 Voronezh, Russia; zvyagin.a@mail.ru; Tel.: +7-919-234-6000; 2 Department of Higher Mathematics, Voronezh State Pedagogical University, Lenina St. 86, 394043 Voronezh, Russia

**Keywords:** viscoelastic fluid, non-linear viscosity, weak solvability, optimal feedback control problem, existence theorem

## Abstract

In this paper we consider the initial–boundary value problem describing the motion of weakly concentrated aqueous polymer solutions. The model involves the regularized Jaumann’s derivative in the rheological relation. Also this model is considered with non-linear viscosity. On the basis of the topological approximation approach to the study of hydrodynamics problems the existence of weak solutions is proved. Also we consider an optimal feedback control problem for this initial–boundary value problem. The existence of an optimal solution minimizing a given performance functional is proved.

## 1. Introduction

In the fluid dynamics theory the motion of an incompressible fluid with a constant density can be described by the equations [1]:

(1) $$\frac{\partial v}{\partial t}+\sum_{i=1}^{n}v_{i}\frac{\partial v}{\partial x_{i}}+\mathrm{grad}p=\mathrm{Div}\sigma +f,$$

(2) divv=0,

where $v\left(x,t\right)=\left(v_{1},\ldots ,v_{n}\right)$ is an unknown velocity field of the fluid; p=p(x,t) is an unknown pressure; f=f(x,t) is the external force; σ is an unknown deviator of the stress tensor. The divergence Divσ of the tensor σ is the vector with coordinates

$$(\sum_{j=1}^{n}\frac{\partial \sigma_{1j}}{\partial x_{j}},\sum_{j=1}^{n}\frac{\partial \sigma_{2j}}{\partial x_{j}},\ldots ,\sum_{j=1}^{n}\frac{\partial \sigma_{nj}}{\partial x_{j}}).$$

System (1) and (2) describes the motion of all kinds of incompressible fluids. However, it is incomplete. As a rule, the additional relation between the deviator of the stress tensor σ and the strain rate tensor $E\left(v\right)={\left(E_{ij}\left(v\right)\right)}_{j=1,\cdots ,n}^{i=1,\cdots ,n}$, $E_{ij}\left(v\right)=\frac{1}{2}(\frac{\partial v_{i}}{\partial x_{j}}+\frac{\partial v_{j}}{\partial x_{i}})$. Such relations are known as constitutive or rheological laws. Choosing a rheological relation we specify a type of fluid (see [2]). Note that this relation should corresponds to the general requirements for a mathematical model. The main of which are the maximum proximity of the results obtained by this relation to the real fluid characteristics at the maximum simplicity of the relation itself.

This paper is devoted to the viscoelastic fluids. The rheological relation for such type of fluids is the following (see [3,4,5,6]):

(3) $$\sigma =2\mu E+2\varkappa \dot{E}.$$

Here μ>0 is the fluid viscosity and ϰ>0 is the retardation time.

The rheological relation of viscoelastic fluids always contain the time parameter. From the mathematical point of view, it is possible to divide them into two groups: differential (coupling the instantaneous stress values with velocity gradient of the fluid) and integral (reflecting the dependence of the fluid stress from the prehistory of flow). In this paper the first group of rheological relations are considered.

Note that the relation (3) contains time derivative of the strain rate tensor. Mathematical studies of this model started with consideration in rheological relation (3) the partial derivative. This mathematical model (by analogy for the solid body model) received the name: Voigt model and have been studied in detail (see, for example [7,8]). Then one began consider the relation (3) with the total derivative. This model received the name of the Kelvin–Voigt model. The mathematical investigation of an initial–boundary value problem for this case is consider in many papers [9,10,11,12] and the solubility of the stationary case of the problem under consideration is proved in [13,14]. The next investigations of this model are connected with consideration in the relation (3) the objective derivative [15]. This leads to the fact that this rheological relation does not depend on the observer, i.e., this relation does not change under Galilean change of variables. The most general view of the objective derivative has the regularized Jaumann’s derivative (see [16]):

(4) $$\begin{array}{c} \frac{DT(t,x)}{Dt}=\frac{dT(t,x)}{dt}+T\left(t,x\right)W_{\rho }\left(t,x\right)-W_{\rho }\left(t,x\right)T\left(t,x\right),\\ W_{\rho }\left(v\right)\left(t,x\right)=\int_{R^{n}}\rho \left(x-y\right)W\left(t,y\right)\;dy, \end{array}$$

where $\rho :R^{n}\to R$ is a smooth function with compact support such that $\int_{R^{n}}\rho \left(y\right)\;dy=1$ and ρ(x)=ρ(y) for *x* and *y* with the same Euclidean norm; $W\left(v\right)={\left(W_{ij}\left(v\right)\right)}_{j=1,\cdots ,n}^{i=1,\cdots ,n}$, $W_{ij}\left(v\right)=\frac{1}{2}\left(\frac{\partial v_{i}}{\partial x_{j}}-\frac{\partial v_{j}}{\partial x_{i}}\right)$ is the vorticity tensor. Note that the rheological law (3) with the regularized Jaumann’s derivative is similar to a particular case of second grade fluids (e.g., see [17,18,19] and the bibliography therein). The weak solvability for this model is proved in [20]. Trajectory, global and pullback attractors for this model are considered in [21]. And finally an optimal feedback control problem for this model is investigated in [22].

At the same time according to Stokes’ hypothesis the stress tensor at a point at a given time is completely determined by the strain rate at the same point and at the same time. However, this relationship does not imply any restrictions associated with linearity, but it is believed that deformation occurring at some other point or at some other point in time prior to the considered one does not affect the value of stresses. The latter circumstance is taken into account in models of nonlinear viscoelastic media. The study of models with nonlinear viscosity, on the one hand, makes it possible to significantly expand the class of studied media, on the other hand, it significantly complicates the mathematical research of such initial–boundary value problems (due to the complexity of the problem). Note that many functions of nonlinear viscosity have been proposed in the literature. At this paper we will consider some of the natural viscosity constraints for real fluids proposed by V.G. Litvinov [23]:

(5) $$\mu =\mu (I_{2}\left(v\right)),$$

where the tensor $I_{2}\left(v\right)$ is defined by the relation $I_{2}{\left(v\right)}^{2}=E\left(v\right):E\left(v\right)$. Here we use the notation $A:B:=\sum_{i,j=1}^{n}a_{ij}b_{ij}$ for arbitrary square matrices $A=(a_{ij})$ and $B=(b_{ij})$ of the same order.

Professor V.G. Litvinov gave examples of such fluids and natural restrictions on the viscosity of the fluid under consideration expressed via the properties of the function $\mu :R^{n}\to R$. This function μ(s) must be continuously differentiable and satisfy the inequalities

(*μ*_1_)
$0<C_{1}\leq \mu \left(s\right)\leq C_{2}<\infty ;$

(*μ*_2_)
$-s\mu^{\prime }\left(s\right)\leq \mu \left(s\right)for\mu^{\prime }\left(s\right)<0;$

(*μ*_3_)
$|s\mu^{\prime }\left(s\right)|\leq C_{3}<\infty .$

Hereinafter, $C_{i}$ denotes various constants. Conditions $\left(\mu_{1}\right)-\left(\mu_{3}\right)$ have a clear physical meaning. Condition $\left(\mu_{1}\right)$ is connected with the existence of limit “Newtonian” viscosities for real fluids; $\left(\mu_{2}\right)-\left(\mu_{3}\right)$ express the law that the shift stresses grow together with the deformation rates. Similar mathematical models with nonlinear viscosity have been considered in many papers (see, for example, Waele–Ostwald model, Norton–Hoff model, Sisko model et al.). In general, many types of function μ have been proposed in the literature, but most of them have been applied to the study of one–dimensional models. In the paper [23] it is shown that the given restrictions on the function μ are natural for real fluids and that the numerical results for this model is very close to the results of experimental studies.

Substituting the right–hand of (3) with nonlinear viscosity (5) and with the regularized Jaumann’s derivative (4) for σ in Equations (1) and (2), we obtain

$$\begin{array}{c} \frac{\partial v}{\partial t}+\sum_{i=1}^{n}v_{i}\frac{\partial v}{\partial x_{i}}-2\mathrm{Div}\left[\mu (I_{2}\left(v\right))E\left(v\right)\right]-\varkappa \frac{\partial \Delta v}{\partial t}-2\varkappa \mathrm{Div}\left[v_{k}\frac{\partial E(v)}{\partial x_{k}}\right]- \end{array}$$

(6) $$\begin{array}{c} -2\varkappa \mathrm{Div}\left[E\left(v\right)W_{\rho }\left(v\right)-W_{\rho }\left(v\right)E\left(v\right)\right]+\mathrm{grad}p=f,\;\left(t,x\right)\in \left(0,T\right)\times \Omega , \end{array}$$

(7) $$\begin{array}{c} \mathrm{div}v=0,\;(t,x)\in (0,T)\times \Omega . \end{array}$$

For the system (6) and (7) we consider the initial–boundary value problem with the initial condition

(8) $$v\left(x,0\right)\;=v_{0}\left(x\right),\;x\in \Omega ,$$

and the boundary condition

(9) $${v|}_{\partial \Omega \times [0,T]}\;=0.$$

The obtained with such rheological relation mathematical model have to satisfy with the experimental data. The experimental data for this mathematical model have been also obtained. Obviously, if a small amount of polymer is added to the water, then the viscosity and the density of the resulting solution practically does not change and remain constant (in contrast to its rheological properties). It is fixed the reduction of friction resistance due to polymer additives. In such fluids the equilibrium state is not established immediately after a change in external conditions. It is established with some delay, which is characterized by the value of the retardation time. This delay explained by the processes of internal rearrangement. A group of scientists carried out experiments and proved that these mathematical model describes the flow of weakly concentrated water solutions of polymers, for example, solutions of polyethyleneoxide and polyacrylamid or solutions of polyacrylamide and guar gum [24,25]. Therefore, the model considered in this paper is also often called the model of aqueous polymers solutions motion.

Our aim is to investigate the weak solvability of this initial–boundary value problem (6)–(9) describing the motion of weakly concentrated aqueous polymer solutions with non-linear viscosity. Also we consider the existence of a feedback control problem for this model and prove the existence of an optimal solution of the problem under consideration minimizing a given bounded performance functional.

## 2. Preliminaries and Main Results

At the beginning we introduce some basic notations and auxiliary assertions.

Denote by $C_{0}^{\infty }\left(\Omega \right)$ the space of smooth functions with compact supports in Ω and values in $R^{n}$. Let V be the set $\left\{v\in C_{0}^{\infty }\left(\Omega \right),\mathrm{Div}v=0\right\}$ and let $V^{0}$ and $V^{1}$ be the closures of V in $L_{2}\left(\Omega \right)$ and $W_{2}^{1}\left(\Omega \right)$, respectively. We also use the space $V^{2}=W_{2}^{2}\left(\Omega \right)\cap V^{1}.$

We introduce a scale of space $V^{\alpha },$α∈R (see [26]). To do this, cosider the Leray projection $\pi :L_{2}\left(\Omega \right)\to V^{0}$ and the operator A=−πΔ defined on $V^{2}$. This operator can be extended to a closed self–adjoint operator in $V^{0}$ (we denote the extension by the same letter). The extended operator *A* is positive and has a compact inverse. Let $0<\lambda_{1}⩽\lambda_{2}⩽\lambda_{3}⩽\ldots ⩽\lambda_{k}⩽\ldots$ be the eigenvalues of A. By Hilbert’s theorem on the spectral decomposition of compact operators, the eigenfunctions $\left\{e_{j}\right\}$ of *A* form an orthonormal basis in $V^{0}$. Put

$$E_{\infty }=\left\{v=\sum_{j=1}^{n}v_{j}e_{j}:v_{j}\in R,n\in N\right\}$$

for the set of finite linear combinations of the vectors $e_{j}$ and define $V^{\alpha },\alpha \in R,$ by the completion of $E_{\infty }$ with respect to the norm

$${∥v∥}_{V^{\alpha }}={\left(\sum_{k=1}^{\infty }\lambda_{k}^{\alpha }{\left|v_{k}\right|}^{2}\right)}^{1/2}.$$

Note that this norm for $\alpha >-\frac{1}{2}$ is equivalent to the norm of space $W_{2}^{\alpha }\left(\Omega \right)$ (see [26]) and in the case of α equals 0,1,3 is equivalent to following norms

$${∥v∥}_{V^{0}}=(\int_{\Omega }v^{2}\left(x\right)\;dx{)}^{1/2};\;{∥v∥}_{V^{1}}=(\int_{\Omega }(\nabla v\left(x\right)):(\nabla v\left(x\right))\;dx{)}^{1/2};$$

$${∥v∥}_{V^{3}}=(\int_{\Omega }(\Delta \nabla v\left(x\right)):(\Delta \nabla v\left(x\right))\;dx{)}^{1/2}.$$

By 〈f,v〉 we denote the value of a functional $f\in V^{-\alpha }$ on a function $v\in V^{\alpha }$. We will need following two functional spaces $E_{1}$ and $E_{2}$:

$$E_{1}=\left\{v:v\in L_{\infty }\left(0,T;V^{1}\right),\;v^{\prime }\in L_{2}\left(0,T;V^{-1}\right)\right\}\;\mathrm{with}\mathrm{norm}$$

$${∥v∥}_{E_{1}}={∥v∥}_{L_{\infty }\left(0,T;V^{1}\right)}+{∥v^{\prime }∥}_{L_{2}\left(0,T;V^{-1}\right)}\;\mathrm{and}$$

$$E_{2}=\left\{v:v\in C(\left[0,T\right],V^{3}),\;v^{\prime }\in L_{2}\left(0,T;V^{3}\right)\right\}\;\mathrm{with}\mathrm{norm}$$

$${∥v∥}_{E_{2}}={∥v∥}_{C(\left[0,T\right],V^{3})}+{∥v^{\prime }∥}_{L_{2}\left(0,T;V^{3}\right)}.$$

Weak solutions of the original initial–boundary value problem will be belong to the space $E_{1}$ and weak solutions of the approximation problem will be belong to the space $E_{2}$. Now we ready to define weak solutions to the problem (6)–(9). Assume that $f\in L_{2}\left(0,T;V^{-1}\right)$ and $v_{0}\in V^{1}.$

**Definition** **1.** *A function v from the space $E_{1}$ is called a weak solution to problem *(6)–(9)* if for any $\varphi \in V^{3}$ and almost all t∈(0,T) it satisfies the equality*

$$\int_{\Omega }\frac{\partial v}{\partial t}\varphi \;dx-\int_{\Omega }\sum_{i,j=1}^{n}v_{i}v_{j}\frac{\partial \varphi_{j}}{\partial x_{i}}\;dx+2\int_{\Omega }\mu \left(I_{2}\left(v\right)\right)E\left(v\right):E\left(\varphi \right)\;dx+\varkappa \int_{\Omega }\nabla \left(\frac{\partial v}{\partial t}\right):\nabla \varphi \;dx-$$

$$-\varkappa \int_{\Omega }\sum_{i,j,k=1}^{n}v_{k}\frac{\partial v_{i}}{\partial x_{j}}\frac{\partial^{2}\varphi_{j}}{\partial x_{i}\partial x_{k}}\;dx-\varkappa \int_{\Omega }\sum_{i,j,k=1}^{n}v_{k}\frac{\partial v_{j}}{\partial x_{i}}\frac{\partial^{2}\varphi_{j}}{\partial x_{i}\partial x_{k}}\;dx+$$

$$+2\varkappa \int_{\Omega }\left(E\left(v\right)W_{\rho }\left(v\right)-W_{\rho }\left(v\right)E\left(v\right)\right):\nabla \varphi \;dx=\langle f,\varphi \rangle$$

*and the initial condition $v\left(0\right)=v_{0}.$*

Our main result provides existence of weak solutions.

**Theorem** **1.** *Let $\Omega \subset R^{n}$, n=2,3, be bounded domain with smooth boundary. Then for any external force $f\in L_{2}\left(0,T;V^{-1}\right)$ and initial condition $v_{0}\in V^{1}$ there exists a weak solution of problem *(6)–(9)*.*

Proof of this Theorem is based on the topological approximation approach used for studying mathematical problems of hydrodynamics (see [27,28]). First, we introduce a family of auxiliary problems which depend on a small parameter ε>0, obtain a priori estimates for solutions, and on the base of the theory of topological degree for maps of the Leray–Schauder type prove the existence of weak solutions to the auxiliary problem. Then, we pass to the limit using appropriate estimates.

## 3. Approximating Problem

Assume that external force $f\in L_{2}\left(0,T;V^{-1}\right)$ and initial condition $v_{0}\in V^{3}.$ We consider the following auxiliary problem: find a function $v\in E_{2}$ satisfies the initial condition $v\left(0\right)=v_{0}$, such that for any $\varphi \in V^{3}$ and a.a. t∈(0,T) the equality holds

(10) $$\begin{array}{c} \varepsilon \int_{\Omega }\nabla \left(\Delta (\frac{\partial v}{\partial t})\right):\nabla \left(\Delta \varphi \right)\;dx+\int_{\Omega }\frac{\partial v}{\partial t}\varphi \;dx-\int_{\Omega }\sum_{i,j=1}^{n}v_{i}v_{j}\frac{\partial \varphi_{j}}{\partial x_{i}}\;dx+\\ +2\int_{\Omega }\mu \left(I_{2}\left(v\right)\right)E\left(v\right):E\left(\varphi \right)\;dx+\varkappa \int_{\Omega }\nabla \left(\frac{\partial v}{\partial t}\right):\nabla \varphi \;dx-\varkappa \int_{\Omega }\sum_{i,j,k=1}^{n}v_{k}\frac{\partial v_{i}}{\partial x_{j}}\frac{\partial^{2}\varphi_{j}}{\partial x_{i}\partial x_{k}}\;dx-\\ -\varkappa \int_{\Omega }\sum_{i,j,k=1}^{n}v_{k}\frac{\partial v_{j}}{\partial x_{i}}\frac{\partial^{2}\varphi_{j}}{\partial x_{i}\partial x_{k}}\;dx+2\varkappa \int_{\Omega }\left(E\left(v\right)W_{\rho }\left(v\right)-W_{\rho }\left(v\right)E\left(v\right)\right):\nabla \varphi \;dx=\langle f,\varphi \rangle . \end{array}$$

Let us first give an operator statement of the problem under consideration. Consider the following operators:

$$J:V^{3}\to V^{-3},\;\langle Jv,\varphi \rangle =\int_{\Omega }v\varphi \;dx,\;v,\varphi \in V^{3};$$

$$A:V^{1}\to V^{-1},\;\langle Av,\varphi \rangle =\int_{\Omega }\nabla v:\nabla \varphi \;dx,\;v,\varphi \in V^{1};$$

$$B_{1}:L_{4}{\left(\Omega \right)}^{n}\to V^{-1},\;\langle B_{1}\left(v\right),\varphi \rangle =\int_{\Omega }\sum_{i,j=1}^{n}v_{i}v_{j}\frac{\partial \varphi_{j}}{\partial x_{i}}\;dx,v\in L_{4}{\left(\Omega \right)}^{n},\;\varphi \in V^{1};$$

$$B_{2}:V^{1}\to V^{-3},\;\langle B_{2}\left(v\right),\varphi \rangle =\int_{\Omega }\sum_{i,j,k=1}^{n}v_{k}\frac{\partial v_{i}}{\partial x_{j}}\frac{\partial^{2}\varphi_{j}}{\partial x_{i}\partial x_{k}}\;dx,\;v\in V^{1},\;\varphi \in V^{3};$$

$$B_{3}:V^{1}\to V^{-3},\;\langle B_{3}\left(v\right),\varphi \rangle =\int_{\Omega }\sum_{i,j,k=1}^{n}v_{k}\frac{\partial v_{j}}{\partial x_{i}}\frac{\partial^{2}\varphi_{j}}{\partial x_{i}\partial x_{k}}\;dx,\;v\in V^{1},\;\varphi \in V^{3};$$

$$B_{4}:V^{1}\to V^{-3},\;\langle B_{4}\left(v\right),\varphi \rangle =\int_{\Omega }\left(E\left(v\right)W_{\rho }\left(v\right)-W_{\rho }\left(v\right)E\left(v\right)\right):\nabla \varphi \;dx,\;v\in V^{1},\varphi \in V^{3};$$

$$N:V^{1}\to V^{-1},\;\langle N\left(v\right),\varphi \rangle =2\int_{\Omega }\mu \left(I_{2}\left(v\right)\right)E\left(v\right):E\left(\varphi \right)\;dx,\;v,\varphi \in V^{1};$$

$$D:V^{3}\to V^{-3},\;\langle Dv,\varphi \rangle =\int_{\Omega }\nabla \left(\Delta v\right):\nabla \left(\Delta \varphi \right)\;dx,\;v,\varphi \in V^{3}.$$

Since $\varphi \in V^{3}$ is arbitrary in (3), this equality is equivalent to the following operator equation:

(11) $$Jv^{\prime }+\varkappa Av^{\prime }+\varepsilon Dv^{\prime }+N\left(v\right)-B_{1}\left(v\right)-\varkappa B_{2}\left(v\right)-\varkappa B_{3}\left(v\right)+2\varkappa B_{4}\left(v\right)=f.$$

Thus a weak solution of the approximating problem is a solution $v\in E_{2}$ of operator Equation (11) satisfying the initial condition $v\left(0\right)=v_{0}$.

We also define the following operators:

$$\begin{array}{c} L:E_{2}\to L_{2}\left(0,T;V^{-3}\right)\times V^{3},\;L\left(v\right)=\left(\left(J+\varkappa A+\varepsilon D\right)v^{\prime }{+N\left(v\right),v|}_{t=0}\right);\\ K:E_{2}\to L_{2}\left(0,T;V^{-3}\right)\times V^{3},\\ K\left(v\right)=\left(-B_{1}\left(v\right)-\varkappa B_{2}\left(v\right)-\varkappa B_{3}\left(v\right)+2\varkappa B_{4}\left(v\right),0\right). \end{array}$$

Thus our auxiliary problem can be rewritten in the following way: find a function $v\in E_{2}$ satisfying the following operator equation:

(12) $$L\left(v\right)+K\left(v\right)=\left(f,v_{0}\right).$$

Now we need following properties of the operators

**Lemma** **1.** *The function Av belongs to $L_{2}(0,T;$$V^{-3})$ for any function $v\in E_{2}$. Also the operator $A:E_{2}\to L_{2}\left(0,T;V^{-3}\right)$ is compact and obeys the estimate:*

$${∥Av∥}_{L_{2}\left(0,T;V^{-3}\right)}⩽C_{4}{∥v∥}_{C(\left[0,T\right],V^{1})}.$$

**Lemma** **2.** *For any function $v\in L_{2}\left(0,T;V^{3}\right)$ the function Dv belongs to $L_{2}\left(0,T;V^{-3}\right),$ the operator $D:L_{2}\left(0,T;V^{3}\right)\to L_{2}\left(0,T;V^{-3}\right)$ is continuous and obeys the estimate:*

$${∥Dv∥}_{L_{2}\left(0,T;V^{-3}\right)}⩽{∥v∥}_{L_{2}\left(0,T;V^{3}\right)}.$$

**Lemma** **3.** *For any function $v\in L_{2}\left(0,T;V^{3}\right)$ the function (J+ϰA+εD)v belongs to $L_{2}\left(0,T;V^{-3}\right)$, the operator $\left(J+\varkappa A+\varepsilon D\right):L_{2}\left(0,T;V^{3}\right)\to L_{2}\left(0,T;V^{-3}\right)$ is continuous, invertible, and obeys the estimate*

(13) $${\varepsilon ∥v∥}_{L_{2}\left(0,T;V^{3}\right)}⩽{∥\left(J+\varkappa A+\varepsilon D\right)v∥}_{L_{2}\left(0,T;V^{-3}\right)}⩽\left(C_{5}+\varepsilon +\varkappa C_{6}\right){∥v∥}_{L_{2}\left(0,T;V^{3}\right)}.$$

*Moreover, the inverse operator ${\left(J+\varkappa A+\varepsilon D\right)}^{-1}:L_{2}\left(0,T;V^{-3}\right)\to L_{2}\left(0,T;V^{3}\right)$ is continuous.*

**Lemma** **4.** *For any function $v\in L_{2}\left(0,T;V^{-1}\right)$ the function (J+ϰA)v belongs to $L_{2}\left(0,T;V^{-3}\right)$, the operator $\left(J+\varkappa A\right):L_{2}\left(0,T;V^{-1}\right)\to L_{2}\left(0,T;V^{-3}\right)$ is continuous and obeys the estimate:*

(14) $$\begin{array}{c} C_{7}{∥v∥}_{L_{2}\left(0,T;V^{-1}\right)}⩽{∥\left(J+\varkappa A\right)v∥}_{L_{2}\left(0,T;V^{-3}\right)}. \end{array}$$

**Lemma** **5.** *For any function $v\in E_{2}$ the function $B_{1}\left(v\right)$ belongs to $L_{2}(0,T;$$V^{-3})$, the mapping $B_{1}:E_{2}\to L_{2}\left(0,T;V^{-3}\right)$ is compact and obeys the estimate:*

(15) $$∥B_{1}{\left(v\right)∥}_{L_{2}\left(0,T;V^{-3}\right)}⩽C_{8}{∥v∥}_{C(\left[0,T\right],V^{1})}^{2}.$$

**Lemma** **6.** *For any function $v\in E_{2}$ the function $B_{i}\left(v\right)$ belongs to $L_{2}(0,T;$$V^{-3})$, $B_{i}:E_{2}\to L_{2}\left(0,T;V^{-3}\right)$, i=2,3, is compact and obeys the estimate:*

(16) $$∥B_{i}{\left(v\right)∥}_{L_{2}\left(0,T;V^{-3}\right)}⩽C_{9}{∥v∥}_{C(\left[0,T\right],V^{1})}^{2}.$$

Proofs of Lemmas 1–6 can be found, for example, in [10].

**Lemma** **7.** *The operator $B_{4}$ has the following properties:*

*1.*  *The operator $B_{4}:V^{1}\to V^{-3}$ is continuous and for any $v\in V^{1}$ obeys the estimate:*
(17) $$∥B_{4}{\left(v\right)∥}_{V^{-3}}⩽C_{10}{∥v∥}_{V^{1}}^{2}.$$
*2.*  *For any function $v\in L_{4}\left(0,T;V^{1}\right)$ we have $B_{4}\left(v\right)\in L_{2}\left(0,T;V^{-3}\right)$ and the mapping $B_{4}:L_{4}\left(0,T;V^{1}\right)\to L_{2}\left(0,T;V^{-3}\right)$ is continuous.*
*3.*  *For any function $v\in E_{2}$ we have $B_{4}\left(v\right)\in L_{2}\left(0,T;V^{-3}\right)$ and the mapping $B_{4}:E_{2}\to L_{2}\left(0,T;V^{-3}\right)$ is compact and obeys the estimate:*
(18) $$∥B_{4}{\left(v\right)∥}_{L_{2}\left(0,T;V^{-3}\right)}⩽C_{11}{∥v∥}_{C(\left[0,T\right],V^{1})}^{2}.$$

**Proof.**  *(1)* We start by estimating E and $W_{\rho }$.

$${∥E\left(v\right)∥}_{L_{2}\left(\Omega \right)}^{2}=\sum_{i,j=1}^{n}{∥E_{ij}\left(v\right)∥}_{L_{2}\left(\Omega \right)}^{2}⩽C_{12}\sum_{i,j=1}^{n}\int_{\Omega }{\left(\frac{\partial v_{i}}{\partial x_{j}}+\frac{\partial v_{j}}{\partial x_{i}}\right)}^{2}\;dx=$$

$$=C_{12}\sum_{i,j=1}^{n}\int_{\Omega }\left(\frac{\partial v_{i}}{\partial x_{j}}\frac{\partial v_{i}}{\partial x_{j}}+2\frac{\partial v_{i}}{\partial x_{j}}\frac{\partial v_{j}}{\partial x_{i}}+\frac{\partial v_{j}}{\partial x_{i}}\frac{\partial v_{j}}{\partial x_{i}}\right)\;dx=$$

$$=C_{12}\sum_{i,j=1}^{n}[\int_{\Omega }\frac{\partial v_{i}}{\partial x_{j}}\frac{\partial v_{i}}{\partial x_{j}}\;dx-2\int_{\Omega }v_{i}\frac{\partial^{2}v_{j}}{\partial x_{i}\partial x_{j}}\;dx+\int_{\Omega }\frac{\partial v_{j}}{\partial x_{i}}\frac{\partial v_{j}}{\partial x_{i}}\;dx]=$$

$$=C_{12}[\int_{\Omega }\nabla v:\nabla v\;dx+\int_{\Omega }\nabla v:\nabla v\;dx]⩽2C_{12}{∥v∥}_{V^{1}}^{2}.$$

Therefore, ${∥E\left(v\right)∥}_{L_{2}\left(\Omega \right)}⩽C_{13}{∥v∥}_{V^{1}}$.

$$\begin{array}{c} ∥{\left(W_{\rho }\right)}_{ij}\left(v\right){∥}_{L_{2}\left(\Omega \right)}⩽{∥{\left(W_{\rho }\right)}_{ij}\left(v\right)∥}_{L_{\infty }\left(\Omega \right)}⩽\\ ⩽\frac{1}{2}\underset{x\in \Omega }{\sup}\left|\int_{\Omega }\rho \left(x-y\right)\left(\frac{\partial v_{i}\left(t,y\right)}{\partial y_{j}}-\frac{\partial v_{j}\left(t,y\right)}{\partial y_{i}}\right)\;dx\right|⩽\\ ⩽\frac{1}{2}\underset{x\in \Omega }{\sup}|\int_{\Omega }-\frac{\partial \rho (x-y)}{\partial y_{j}}v_{i}\left(t,y\right)+\frac{\partial \rho (x-y)}{\partial y_{i}}v_{j}{\left(t,y\right)\;dx|⩽∥grad\rho ∥}_{L_{2}\left(\Omega \right)}{∥v\left(t\right)∥}_{L_{2}\left(\Omega \right)}. \end{array}$$

By definition, for any $v\in V^{1},\varphi \in V^{3}$ we have

$$\begin{array}{c} \left|\langle B_{4}\left(v\right),\varphi \rangle \right|=\left|\int_{\Omega }\left(E\left(v\right)W_{\rho }\left(v\right)-W_{\rho }\left(v\right)E\left(v\right)\right):\nabla \varphi \;dx\right|⩽\\ ⩽C_{14}{[∥E\left(v\right)∥}_{L_{2}\left(\Omega \right)}∥W_{\rho }{\left(v\right)∥}_{L_{2}\left(\Omega \right)}+∥W_{\rho }{\left(v\right)∥}_{L_{2}\left(\Omega \right)}{∥E\left(v\right)∥}_{L_{2}\left(\Omega \right)}]{∥\nabla \varphi ∥}_{C{\left(\Omega \right)}^{n}}⩽\\ C_{15}{∥v∥}_{V^{1}}^{2}{∥\varphi ∥}_{V^{3}}. \end{array}$$

This yields the estimate (17).

Now prove that the operator $B_{4}$ continuous. For any $v^{m},v^{0}\in V^{1}$ we have:

$$\begin{array}{c} |\langle B_{4}\left(v^{m}\right),\varphi \rangle -\langle B_{4}\left(v^{0}\right),\varphi \rangle |=|\int_{\Omega }(E\left(v^{m}\right)W_{\rho }\left(v^{m}\right)-W_{\rho }\left(v^{m}\right)E\left(v^{m}\right)):\nabla \varphi \;dx-\\ -\int_{\Omega }(E\left(v^{0}\right)W_{\rho }\left(v^{0}\right)-W_{\rho }\left(v^{0}\right)E\left(v^{0}\right)):\nabla \varphi \;dx|⩽C_{16}|\int_{\Omega }E\left(v^{m}\right)W_{\rho }\left(v^{m}\right)-\\ -W_{\rho }\left(v^{m}\right)E\left(v^{m}\right)-E\left(v^{0}\right)W_{\rho }\left(v^{0}\right)+W_{\rho }\left(v^{0}\right)E\left(v^{0}\right)\;dx|{∥\varphi ∥}_{V^{3}}⩽\\ ⩽C_{16}|\int_{\Omega }E\left(v^{m}\right)(W_{\rho }\left(v^{m}\right)-W_{\rho }\left(v^{0}\right))+(E\left(v^{m}\right)-E\left(v^{0}\right))W_{\rho }\left(v^{0}\right)-\\ -W_{\rho }\left(v^{m}\right)(E\left(v^{m}\right)-E\left(v^{0}\right))-(W_{\rho }\left(v^{m}\right)-W_{\rho }\left(v^{0}\right))E\left(v^{0}\right)\;dx|{∥\varphi ∥}_{V^{3}}⩽\\ ⩽C_{16}[∥E\left(v^{m}\right){∥}_{L_{2}\left(\Omega \right)}∥W_{\rho }\left(v^{m}-v^{0}\right){∥}_{L_{2}\left(\Omega \right)}+{∥E\left(v^{m}-v^{0}\right)∥}_{L_{2}\left(\Omega \right)}\times \\ \times ∥W_{\rho }\left(v^{0}\right){∥}_{L_{2}\left(\Omega \right)}+∥W_{\rho }\left(v^{m}\right){∥}_{L_{2}\left(\Omega \right)}∥E\left(v^{m}-v^{0}\right){∥}_{L_{2}\left(\Omega \right)}+{∥W_{\rho }\left(v^{m}-v^{0}\right)∥}_{L_{2}\left(\Omega \right)}\times \\ \times ∥E\left(v^{0}\right){∥}_{L_{2}\left(\Omega \right)}{]∥\varphi ∥}_{V^{3}}⩽C_{17}[∥v^{m}{∥}_{V^{1}}∥v^{m}-v^{0}{∥}_{V^{1}}+∥v^{m}-v^{0}{∥}_{V^{1}}{∥v^{0}∥}_{V^{1}}+\\ +∥v^{m}{∥}_{V^{1}}∥v^{m}-v^{0}{∥}_{V^{1}}+∥v^{m}-v^{0}{∥}_{V^{1}}∥v^{0}{∥}_{V^{1}}]{∥\varphi ∥}_{V^{3}}⩽\\ ⩽C_{18}(∥v^{m}{∥}_{V^{1}}+∥v^{0}{∥}_{V^{1}})∥v^{m}-v^{0}{∥}_{V^{1}}{∥\varphi ∥}_{V^{3}}. \end{array}$$

Thus we get

(19) $${∥B_{4}\left(v^{m}\right)-B_{4}\left(v^{0}\right)∥}_{V^{-3}}⩽C_{18}\left({∥v^{m}∥}_{V^{1}}+{∥v^{0}∥}_{V^{1}}\right){∥v^{m}-v^{0}∥}_{V^{1}}.$$

Let the sequence $\left\{v^{m}\right\}\subset V^{1}$ converge to some limiting function $v^{0}\in V^{1}.$ Then the continuity of the mapping $B_{4}:V^{1}\to V^{-3}$ follows from the previous inequality.

*(2)* Let $v\in L_{4}\left(0,T;V^{1}\right).$ Then from (17) for almost all t∈(0,T) we get the estimate

$$∥B_{4}{\left(v\right)\left(t\right)∥}_{V^{-3}}⩽C_{10}{∥v\left(t\right)∥}_{V^{1}}^{2}.$$

Squaring this estimate and integrating with respect to *t* from 0 to *T*, we get

$$\int_{0}^{T}∥B_{4}{\left(v\right)\left(t\right)∥}_{V^{-3}}^{2}dt⩽C_{10}^{2}\int_{0}^{T}{∥v\left(t\right)∥}_{V^{1}}^{4}dt=C_{10}^{2}{∥v∥}_{L_{4}\left(0,T;V^{1}\right)}^{4}.$$

This yields $B_{4}\left(v\right)\in L_{2}\left(0,T;V^{-3}\right).$

Now prove that the continuity of the mapping $B_{4}:L_{4}\left(0,T;V^{1}\right)\to L_{2}(0,T;$$V^{-3}).$

Let the sequence $\left\{v^{m}\right\}\subset L_{4}\left(0,T;V^{1}\right)$ converge to some limit $v^{0}\in L_{4}(0,T;$$V^{1}).$ Square the inequality (19) and integrate with respect to *t* from 0 to T. Using the Hölder inequality, we obtain

$$\begin{array}{c} {∥B_{4}\left(v^{m}\right)-B_{4}\left(v^{0}\right)∥}_{L_{2}\left(0,T;V^{-3}\right)}⩽C_{18}^{2}{∥v^{m}-v^{0}∥}_{L_{4}\left(0,T;V^{1}\right)}\times \\ \;\;\;\;\;\;\;\;\;\;\;\;\;\;\;\times {\left(\sum_{i=0}^{4}\frac{4!}{i!(4-i)!}{∥v^{m}∥}_{L_{4}\left(0,T;V^{1}\right)}^{i}{∥v^{0}∥}_{L_{4}\left(0,T;V^{1}\right)}^{4-i}\right)}^{\frac{1}{4}}. \end{array}$$

We get that the left–hand side tends to zero. So we prove that $B_{4}:L_{4}\left(0,T;V^{1}\right)\to L_{2}\left(0,T;V^{-3}\right)$ is continuous.

*(3)* Finally, to prove (3) part we use the following Theorem

**Theorem** **2** (Simon, [29])**.** *Let X⊂E⊂Y be Banach spaces, the embedding X⊂E be compact, and the embedding E⊂Y be continuous. Let $F\subset L_{p}\left(0,T;X\right),\;1⩽p⩽\infty .$ Suppose that for any f∈F its generalized derivative in the space $D^{\prime }\left(0,T;Y\right)$ belongs to $L_{r}\left(0,T;Y\right),\;1⩽r⩽\infty .$ Then, let*

*1.*  *the set F be bounded in $L_{p}\left(0,T;X\right),$*
*2.*  *the set $\left\{f^{\prime }:\;f\in F\right\}$ be bounded in $L_{r}\left(0,T;Y\right).$*

*Then the set F is relatively compact in $L_{p}\left(0,T;E\right)$ for p<∞, and the set F is relatively compact in C([0,T],E) for p=∞ and r>1.*

In our case let

$$\begin{array}{c} X=V^{3},\;E=V^{1},\;Y=V^{0},\\ F=\{v:v\in L_{4}\left(0,T;V^{3}\right);v^{\prime }\in L_{2}\left(0,T;V^{0}\right)\}. \end{array}$$

We have the compact embedding $V^{3}\subset V^{1}$. So why *F* is embedded into $L_{4}\left(0,T;V^{1}\right)$ compactly. Also we have

$$C\left(\left[0,T\right],V^{3}\right)\subset L_{4}\left(0,T;V^{3}\right),\;L_{2}\left(0,T;V^{3}\right)\subset L_{2}\left(0,T;V^{0}\right).$$

that gives us that $E_{2}\subset F$. Finally, we have

$$E_{2}\subset F\subset L_{4}\left(0,T;V^{1}\right)\overset{B_{4}}{\to }L_{2}\left(0,T;V^{-3}\right).$$

Here the first embedding is continuous, the second embedding is compact and the mapping $B_{4}$ is continuous. Thus, for any function $v\in E_{2}$ we see that the function $B_{4}\left(v\right)\in L_{2}\left(0,T;V^{-3}\right),$ and the mapping $B_{4}:E_{2}\to L_{2}\left(0,T;V^{-3}\right)$ is compact.

Now prove the estimate (18). By (17), the estimate

$$∥B_{4}{\left(v\right)\left(t\right)∥}_{V^{-3}}⩽C_{10}{∥v\left(t\right)∥}_{V^{1}}^{2}.$$

holds for all t∈[0,T]. Squaring it and integrating with respect to *t* from 0 to *T*, we get

$$\int_{0}^{T}∥B_{4}{\left(v\right)\left(t\right)∥}_{V^{-3}}^{2}dt⩽C_{10}^{2}\int_{0}^{T}{∥v\left(t\right)∥}_{V^{1}}^{4}dt⩽C_{10}^{2}T{∥v∥}_{C(\left[0,T\right],V^{1})}^{4}.$$

This yields the required estimate (18). □

**Lemma** **8.** *If the function μ satisfies conditions $\left(\mu_{1}\right)-\left(\mu_{3}\right)$ for any function v from $C(\left[0,T\right],V^{3})$ the function N(v) belongs to $L_{2}\left(0,T;V^{-3}\right)$. The map $N:C\left(\left[0,T\right],V^{3}\right)\to L_{2}\left(0,T;V^{-3}\right)$ is bounded, continuous, monotone and the following inequality holds:*

(20) $$\langle N\left(v\right),v\rangle \leq C_{19}{∥v∥}_{C(\left[0,T\right],V^{3})}^{4}-C_{20},$$

*with constants $C_{19}$ and $C_{20}$ independent of v.*

The proof of this Lemma can be found in [30].

**Lemma** **9.** *The operators L and K have the following properties*

*1*.  *The operator $L:E_{2}\to L_{2}\left(0,T;V^{-3}\right)\times V^{3}$ is invertible and the inverse operator is continuous.*
*2*.  *The operator $K:E_{2}\to L_{2}\left(0,T;V^{-3}\right)\times V^{3}$ is compact.*

**Proof.**  *(1)* To prove that the operator *L* is invertible it is sufficient to use Theorem 1.1 from [31] (Chapter 4). Since $N:V^{1}\to V^{-1}$ is continuous and monotone then all conditions of this Theorem 1.1 are hold. Applying this Theorem 1.1 shows that for each $\left(f,v_{0}\right)$ there exists solution $v\in L_{2}\left(0,T;V^{3}\right)$ and, hence, $v\in E_{2}$. Thus, the operator *L* is inverse.

*(2)* The complete continuity of the operator

$$\begin{array}{c} K:E_{2}\to L_{2}\left(0,T;V^{-3}\right)\times V^{3},\\ K\left(v\right)=\left(-B_{1}\left(v\right)-\varkappa B_{2}\left(v\right)-\varkappa B_{3}\left(v\right)+2\varkappa B_{4}\left(v\right),0\right) \end{array}$$

follows from the compactness of the operators $A:E_{2}\to L_{2}\left(0,T;V^{-3}\right)$ Lemma 1; $B_{1}:E_{2}\to L_{2}\left(0,T;V^{-3}\right)$ Lemma 5; $B_{2}:E_{2}\to L_{2}\left(0,T;V^{-3}\right)$ Lemma 6; $B_{3}:E_{2}\to L_{2}\left(0,T;V^{-3}\right)$ Lemma 6; $B_{4}:E_{2}\to L_{2}\left(0,T;V^{-3}\right)$ Lemma 7. □

## 4. A Priori Estimate

Along with Equation (12) consider the following family of operator equations:

(21) $$L\left(v\right)+\lambda K\left(v\right)=\lambda \left(f,v_{0}\right),\;\lambda \in \left[0,1\right],$$

which coincides with the Equation (12) for λ=1.

**Theorem** **3.** *If $v\in E_{2}$ is a solution of operator Equation *(21)* for some λ∈[0,1], then the following estimate holds:*

(22) $$\begin{array}{c} {\varepsilon ∥v∥}_{C(\left[0,T\right],V^{3})}^{2}⩽C_{21}+2\varepsilon {∥v_{0}∥}_{V^{3}}^{2}, \end{array}$$

(23) $$\begin{array}{c} {\varkappa ∥v∥}_{C(\left[0,T\right],V^{1})}^{2}⩽C_{21}+2\varepsilon {∥v_{0}∥}_{V^{3}}^{2}, \end{array}$$

*where*

$$C_{21}=\frac{4T}{\varkappa }{∥f∥}_{L_{2}\left(0,T;V^{-1}\right)}^{2}+2∥v_{0}{∥}_{V^{0}}^{2}+2\varkappa {∥v_{0}∥}_{V^{1}}^{2}.$$

**Proof.**  Let $v\in E_{2}$ be a solution of (21). Then for any $\varphi \in V^{3}$ and almost all t∈(0,T) the following equation holds:

(24) $$\begin{array}{c} \int_{\Omega }v^{\prime }\left(t\right)\varphi \;dx-\lambda \int_{\Omega }\sum_{i,j=1}^{n}v_{i}\left(t\right)v_{j}\left(t\right)\frac{\partial \varphi_{j}}{\partial x_{i}}\;dx+2\lambda \int_{\Omega }\mu \left(I_{2}\left(v\left(t\right)\right)\right)E\left(v\left(t\right)\right):E\left(\varphi \right)\;dx+\\ +\varepsilon \int_{\Omega }\nabla \left(\Delta v^{\prime }\left(t\right)\right):\nabla \left(\Delta \varphi \right)\;dx+\varkappa \int_{\Omega }\nabla \left(v^{\prime }\left(t\right)\right):\nabla \varphi \;dx-\\ -\lambda \varkappa \int_{\Omega }\sum_{i,j,k=1}^{n}v_{k}\left(t\right)\frac{\partial v_{i}\left(t\right)}{\partial x_{j}}\frac{\partial^{2}\varphi_{j}}{\partial x_{i}\partial x_{k}}\;dx-\lambda \varkappa \int_{\Omega }\sum_{i,j,k=1}^{n}v_{k}\left(t\right)\frac{\partial v_{j}\left(t\right)}{\partial x_{i}}\frac{\partial^{2}\varphi_{j}}{\partial x_{i}\partial x_{k}}\;dx+\\ +2\lambda \varkappa \int_{\Omega }\left(E\left(v\right)W_{\rho }\left(v\right)-W_{\rho }\left(v\right)E\left(v\right)\right):\nabla \varphi \;dx=\lambda \langle f\left(t\right),\varphi \rangle \end{array}$$

Note that

$$\int_{\Omega }\sum_{i,j,k=1}^{n}v_{k}\left(t\right)\frac{\partial v_{i}\left(t\right)}{\partial x_{j}}\frac{\partial^{2}\varphi_{j}}{\partial x_{i}\partial x_{k}}\;dx+\int_{\Omega }\sum_{i,j,k=1}^{n}v_{k}\left(t\right)\frac{\partial v_{j}\left(t\right)}{\partial x_{i}}\frac{\partial^{2}\varphi_{j}}{\partial x_{i}\partial x_{k}}\;dx=$$

$$=2\int_{\Omega }\sum_{i,j,k=1}^{n}v_{k}\left(t\right)E_{ij}\left(v\right)\left(t\right)\frac{\partial^{2}\varphi_{j}}{\partial x_{i}\partial x_{k}}\;dx=-2\int_{\Omega }\sum_{i,j,k=1}^{n}v_{k}\left(t\right)\frac{\partial E_{ij}\left(v\right)\left(t\right)}{\partial x_{k}}\frac{\partial \varphi_{j}}{\partial x_{i}}\;dx-$$

$$-2\int_{\Omega }\sum_{i,j,k=1}^{n}\frac{\partial v_{k}\left(t\right)}{\partial x_{k}}E_{ij}\left(v\right)\left(t\right)\frac{\partial \varphi_{j}}{\partial x_{i}}\;dx=-2\int_{\Omega }\sum_{i,j,k=1}^{n}v_{k}\left(t\right)\frac{\partial E_{ij}\left(v\right)\left(t\right)}{\partial x_{k}}\frac{\partial \varphi_{j}}{\partial x_{i}}\;dx-$$

$$-2\int_{\Omega }\mathrm{div}v\left(t\right)\sum_{i,j=1}^{n}E_{ij}\left(v\right)\left(t\right)\frac{\partial \varphi_{j}}{\partial x_{i}}\;dx=-2\int_{\Omega }\sum_{i,j,k=1}^{n}v_{k}\left(t\right)\frac{\partial E_{ij}\left(v\right)\left(t\right)}{\partial x_{k}}\frac{\partial \varphi_{j}}{\partial x_{i}}\;dx.$$

Then (24) can be rewritten in the form

$$\int_{\Omega }v^{\prime }\left(t\right)\varphi \;dx-\lambda \int_{\Omega }\sum_{i,j=1}^{n}v_{i}\left(t\right)v_{j}\left(t\right)\frac{\partial \varphi_{j}}{\partial x_{i}}\;dx+2\lambda \int_{\Omega }\mu \left(I_{2}\left(v\left(t\right)\right)\right)E\left(v\left(t\right)\right):E\left(\varphi \right)\;dx+$$

$$+\varepsilon \int_{\Omega }\nabla \left(\Delta v^{\prime }\left(t\right)\right):\nabla \left(\Delta \varphi \right)\;dx+\varkappa \int_{\Omega }\nabla \left(v^{\prime }\left(t\right)\right):\nabla \varphi \;dx+$$

$$+2\lambda \varkappa \int_{\Omega }\sum_{i,j,k=1}^{n}v_{k}\left(t\right)\frac{\partial E_{ij}\left(v\right)\left(t\right)}{\partial x_{k}}\frac{\partial \varphi_{j}}{\partial x_{i}}\;dx+2\lambda \varkappa \int_{\Omega }\left(E\left(v\right)W_{\rho }\left(v\right)-W_{\rho }\left(v\right)E\left(v\right)\right):\nabla \varphi \;dx=$$

=λ〈f(t),φ〉.

Since the last equation holds for all $\varphi \in V^{3},$ it holds for φ=v as well:

(25) $$\begin{array}{c} \int_{\Omega }v^{\prime }\left(t\right)v\left(t\right)dx-\lambda \int_{\Omega }\sum_{i,j=1}^{n}v_{i}\left(t\right)v_{j}\left(t\right)\frac{\partial v_{j}\left(t\right)}{\partial x_{i}}\;dx+2\lambda \int_{\Omega }\mu \left(I_{2}\left(v\left(t\right)\right)\right)E\left(v\left(t\right)\right):E\left(v\left(t\right)\right)\;dx+\\ +\varepsilon \int_{\Omega }\nabla \left(\Delta v^{\prime }\left(t\right)\right):\nabla \left(\Delta v(t)\right)\;dx+\varkappa \int_{\Omega }\nabla \left(v^{\prime }\left(t\right)\right):\nabla v\left(t\right)dx+\\ +2\lambda \varkappa \int_{\Omega }\sum_{i,j,k=1}^{n}v_{k}\left(t\right)\frac{\partial E_{ij}\left(v\right)\left(t\right)}{\partial x_{k}}\frac{\partial v_{j}\left(t\right)}{\partial x_{i}}\;dx+\\ +2\lambda \varkappa \int_{\Omega }\left(E\left(v\right)W_{\rho }\left(v\right)-W_{\rho }\left(v\right)E\left(v\right)\right):\nabla v\;dx=\lambda \langle f\left(t\right),v\rangle . \end{array}$$

We reduce the terms on the left-hand side of the Equation (25) in the following way:

$$\int_{\Omega }v^{\prime }\left(t\right)v\left(t\right)dx=\frac{1}{2}\int_{\Omega }\frac{\partial \left(v(t)v(t)\right)}{\partial t}\;dx=\frac{1}{2}\frac{d}{dt}\int_{\Omega }v\left(t\right)v\left(t\right)dx=\frac{1}{2}\frac{d}{dt}{∥v\left(t\right)∥}_{V^{0}}^{2};$$

$$\langle N\left(v\right),v\left(t\right)\rangle =2\int_{\Omega }\mu \left(I_{2}\left(v\right)\right)E\left(v\right):E\left(v\right)\;dx\geq C_{22}\int_{\Omega }E\left(v\right):E\left(v\right)\;dx\geq C_{22}{∥v∥}_{V^{1}}^{2};$$

$$\varepsilon \int_{\Omega }\nabla \left(\Delta v^{\prime }\left(t\right)\right):\nabla \left(\Delta v(t)\right)\;dx=\frac{\varepsilon }{2}\int_{\Omega }\frac{\partial }{\partial t}(\nabla \left(\Delta v\left(t\right)\right):\nabla \left(\Delta v(t)\right))\;dx=$$

$$=\frac{\varepsilon }{2}\frac{d}{dt}\int_{\Omega }\nabla \left(\Delta v\left(t\right)\right):\nabla \left(\Delta v(t)\right)\;dx=\frac{\varepsilon }{2}\frac{d}{dt}{∥v\left(t\right)∥}_{V^{3}}^{2};$$

$$\varkappa \int_{\Omega }\nabla \left(v^{\prime }\left(t\right)\right):\nabla v\left(t\right)dx=\frac{\varkappa }{2}\int_{\Omega }\frac{\partial }{\partial t}(\nabla v\left(t\right):\nabla v\left(t\right))\;dx=\frac{\varkappa }{2}\frac{d}{dt}{∥v\left(t\right)∥}_{V^{1}}^{2};$$

$$\int_{\Omega }\sum_{i,j=1}^{n}v_{i}\left(t\right)v_{j}\left(t\right)\frac{\partial v_{j}\left(t\right)}{\partial x_{i}}\;dx=\frac{1}{2}\int_{\Omega }\sum_{i,j=1}^{n}v_{i}\left(t\right)\frac{\partial \left(v_{j}\left(t\right)v_{j}\left(t\right)\right)}{\partial x_{i}}\;dx=$$

$$=-\frac{1}{2}\int_{\Omega }\sum_{i,j=1}^{n}\frac{\partial v_{i}\left(t\right)}{\partial x_{i}}v_{j}\left(t\right)v_{j}\left(t\right)\;dx=-\frac{1}{2}\int_{\Omega }\mathrm{div}v\left(t\right)\sum_{j=1}^{n}v_{j}\left(t\right)v_{j}\left(t\right)\;dx=0;$$

$$\int_{\Omega }(E\left(v\right)W_{\rho }\left(v\right)-W_{\rho }\left(v\right)E\left(v\right)):\nabla v\;dx=\frac{1}{2}\int_{\Omega }(E\left(v\right)W_{\rho }\left(v\right)-W_{\rho }\left(v\right)E\left(v\right)):$$

$$:(E\left(v\right)+W\left(v\right))\;dx=\frac{1}{2}\int_{\Omega }(E\left(v\right)W_{\rho }\left(v\right)-W_{\rho }\left(v\right)E\left(v\right)):E\left(v\right)\;dx+$$

$$+\frac{1}{2}\int_{\Omega }(E\left(v\right)W_{\rho }\left(v\right)-W_{\rho }\left(v\right)E\left(v\right)):W\left(v\right)\;dx=\frac{1}{2}\sum_{i,j,k=1}^{n}\int_{\Omega }(E_{ij}{\left(W_{\rho }\right)}_{jk}E_{ik}-$$

$$-{\left(W_{\rho }\right)}_{jk}E_{ki}E_{ji})\;dx+\frac{1}{2}\sum_{i,j,k=1}^{n}\int_{\Omega }(E_{ij}{\left(W_{\rho }\right)}_{jk}W_{ik}-{\left(W_{\rho }\right)}_{kj}E_{ji}W_{ki})\;dx=$$

$$=\frac{1}{2}\sum_{i,j,k=1}^{n}\int_{\Omega }E_{ij}{\left(W_{\rho }\right)}_{jk}E_{ik}-E_{ij}{\left(W_{\rho }\right)}_{jk}E_{ik}\;dx+\frac{1}{2}\sum_{i,j,k=1}^{n}\int_{\Omega }E_{ij}{\left(W_{\rho }\right)}_{jk}W_{ik}-$$

$$-E_{ij}{\left(W_{\rho }\right)}_{jk}W_{ik}\;dx=0;$$

$$2\int_{\Omega }\sum_{i,j,k=1}^{n}v_{k}\left(t\right)\frac{\partial E_{ij}\left(v\right)\left(t\right)}{\partial x_{k}}\frac{\partial v_{j}\left(t\right)}{\partial x_{i}}\;dx=\int_{\Omega }\sum_{i,j,k=1}^{n}v_{k}\left(t\right)\frac{\partial E_{ij}\left(v\right)\left(t\right)}{\partial x_{k}}\frac{\partial v_{j}\left(t\right)}{\partial x_{i}}\;dx+$$

$$+\int_{\Omega }\sum_{i,j,k=1}^{n}v_{k}\left(t\right)\frac{\partial E_{ij}\left(v\right)\left(t\right)}{\partial x_{k}}\frac{\partial v_{i}\left(t\right)}{\partial x_{j}}\;dx=2\int_{\Omega }\sum_{i,j,k=1}^{n}v_{k}\left(t\right)\frac{\partial E_{ij}\left(v\right)\left(t\right)}{\partial x_{k}}E_{ij}\left(v\right)\left(t\right)dx=$$

$$=\int_{\Omega }\sum_{i,j,k=1}^{n}v_{k}\left(t\right)\frac{\partial \left(E_{ij}\left(v\right)\left(t\right)E_{ij}\left(v\right)\left(t\right)\right)}{\partial x_{k}}\;dx=-\int_{\Omega }\sum_{k=1}^{n}\frac{\partial v_{k}\left(t\right)}{\partial x_{k}}\sum_{i,j=1}^{n}E_{ij}\left(v\right)\left(t\right)\times E_{ij}\left(v\right)\left(t\right)dx=$$

$$=-\int_{\Omega }\mathrm{div}v\left(t\right)\sum_{i,j=1}^{n}E_{ij}\left(v\right)\left(t\right)E_{ij}\left(v\right)\left(t\right)dx=0.$$

Here we take into account that the strain-rate tensor E(v) is symmetric and tensors $W_{\rho }\left(v\right)$ and W(v) are skew-symmetric. Hence the Equation (25) can be rewritten in the following form:

$$\frac{1}{2}\frac{d}{dt}{∥v\left(t\right)∥}_{V^{0}}^{2}+\frac{\varepsilon }{2}\frac{d}{dt}{∥v\left(t\right)∥}_{V^{3}}^{2}+\frac{\varkappa }{2}\frac{d}{dt}{∥v\left(t\right)∥}_{V^{1}}^{2}+\lambda C_{22}{∥v\left(t\right)∥}_{V^{1}}^{2}\leq \lambda \langle f\left(t\right),v\left(t\right)\rangle .$$

Estimating of the right-hand side of the last equation from above as follows

$$\lambda \langle f\left(t\right),v\left(t\right)\rangle ⩽\lambda \left|\langle f(t),v(t)\rangle \right|⩽{\lambda ∥f\left(t\right)∥}_{V^{-1}}{∥v\left(t\right)∥}_{V^{1}}⩽{∥f\left(t\right)∥}_{V^{-1}}{∥v\left(t\right)∥}_{V^{1}},$$

and the left-hand side from below as follows

$$\frac{1}{2}\frac{d}{dt}{∥v\left(t\right)∥}_{V^{0}}^{2}+\frac{\varepsilon }{2}\frac{d}{dt}{∥v\left(t\right)∥}_{V^{3}}^{2}+\frac{\varkappa }{2}\frac{d}{dt}{∥v\left(t\right)∥}_{V^{1}}^{2}⩽$$

$$⩽\frac{1}{2}\frac{d}{dt}{∥v\left(t\right)∥}_{V^{0}}^{2}+\frac{\varepsilon }{2}\frac{d}{dt}{∥v\left(t\right)∥}_{V^{3}}^{2}+\frac{\varkappa }{2}\frac{d}{dt}{∥v\left(t\right)∥}_{V^{1}}^{2}+\lambda C_{22}{∥v\left(t\right)∥}_{V^{1}}^{2},$$

we see that

$$\frac{1}{2}\frac{d}{dt}{∥v\left(t\right)∥}_{V^{0}}^{2}+\frac{\varepsilon }{2}\frac{d}{dt}{∥v\left(t\right)∥}_{V^{3}}^{2}+\frac{\varkappa }{2}\frac{d}{dt}{∥v\left(t\right)∥}_{V^{1}}^{2}⩽{∥f\left(t\right)∥}_{V^{-1}}{∥v\left(t\right)∥}_{V^{1}}.$$

Integrating the last inequality with respect to *t* from 0 to τ, where τ∈[0,T], we obtain

$$\frac{1}{2}{∥v\left(\tau \right)∥}_{V^{0}}^{2}-\frac{1}{2}∥v_{0}{∥}_{V^{0}}^{2}+\frac{\varepsilon }{2}{∥v\left(\tau \right)∥}_{V^{3}}^{2}-\frac{\varepsilon }{2}∥v_{0}{∥}_{V^{3}}^{2}+\frac{\varkappa }{2}{∥v\left(\tau \right)∥}_{V^{1}}^{2}-$$

$$-\frac{\varkappa^{2}}{2}∥v_{0}{∥}_{V^{1}}^{2}⩽\int_{0}^{\tau }{∥f\left(t\right)∥}_{V^{-1}}{∥v\left(t\right)∥}_{V^{1}}\;dt.$$

The right-hand side of the last inequality can be estimated in the following way:

$$\int_{0}^{\tau }{∥f\left(t\right)∥}_{V^{-1}}{∥v\left(t\right)∥}_{V^{1}}\;dt⩽\max_{t\in [0,\tau ]}{∥v\left(t\right)∥}_{V^{1}}\int_{0}^{\tau }{∥f\left(t\right)∥}_{V^{-1}}dt⩽$$

$$⩽\max_{t\in [0,T]}{∥v\left(t\right)∥}_{V^{1}}\int_{0}^{T}{∥f\left(t\right)∥}_{V^{-1}}dt⩽\sqrt{T}\max_{t\in [0,T]}{∥v\left(t\right)∥}_{V^{1}}{\left(\int_{0}^{T}{∥f\left(t\right)∥}_{V^{-1}}^{2}dt\right)}^{\frac{1}{2}}=$$

$$=\sqrt{T}{∥v∥}_{C(\left[0,T\right],V^{1})}{∥f∥}_{L_{2}\left(0,T;V^{-1}\right)}⩽\frac{\varkappa }{4}{∥v∥}_{C(\left[0,T\right],V^{1})}^{2}+\frac{T}{\varkappa }{∥f∥}_{L_{2}\left(0,T;V^{-1}\right)}^{2}.$$

Here we used the Hölder inequality and the Cauchy inequality:

$$bc⩽\frac{\delta b^{2}}{2}+\frac{c^{2}}{2\delta }$$

for $\delta =\frac{\varkappa }{2}.$ Thus, we get

$$\begin{array}{c} \frac{1}{2}{∥v\left(\tau \right)∥}_{V^{0}}^{2}+\frac{\varepsilon }{2}{∥v\left(\tau \right)∥}_{V^{3}}^{2}+\frac{\varkappa }{2}{∥v\left(\tau \right)∥}_{V^{1}}^{2}⩽\\ ⩽\frac{T}{\varkappa }{∥f∥}_{L_{2}\left(0,T;V^{-1}\right)}^{2}+\frac{\varkappa }{4}{∥v∥}_{C(\left[0,T\right],V^{1})}^{2}+\frac{1}{2}∥v_{0}{∥}_{V^{0}}^{2}+\frac{\varepsilon }{2}∥v_{0}{∥}_{V^{3}}^{2}+\frac{\varkappa }{2}{∥v_{0}∥}_{V^{1}}^{2}. \end{array}$$

Taking into account that $\frac{1}{2}{∥v\left(\tau \right)∥}_{V^{0}}^{2}⩾0,$ we have

$$\begin{array}{c} \frac{\varepsilon }{2}{∥v\left(\tau \right)∥}_{V^{3}}^{2}+\frac{\varkappa }{2}{∥v\left(\tau \right)∥}_{V^{1}}^{2}⩽\\ ⩽\frac{T}{\varkappa }{∥f∥}_{L_{2}\left(0,T;V^{-1}\right)}^{2}+\frac{\varkappa }{4}{∥v∥}_{C(\left[0,T\right],V^{1})}^{2}+\frac{1}{2}∥v_{0}{∥}_{V^{0}}^{2}+\frac{\varepsilon }{2}∥v_{0}{∥}_{V^{3}}^{2}+\frac{\varkappa }{2}{∥v_{0}∥}_{V^{1}}^{2}. \end{array}$$

Hence, since ${∥v\left(\tau \right)∥}_{V^{3}}^{2}$ and ${∥v\left(\tau \right)∥}_{V^{1}}^{2}$ are positive we get the estimates

$$\begin{array}{c} \frac{\varepsilon }{2}{∥v\left(\tau \right)∥}_{V^{3}}^{2}⩽\frac{T}{\varkappa }{∥f∥}_{L_{2}\left(0,T;V^{-1}\right)}^{2}+\frac{\varkappa }{4}{∥v∥}_{C(\left[0,T\right],V^{1})}^{2}+\frac{1}{2}∥v_{0}{∥}_{V^{0}}^{2}+\frac{\varepsilon }{2}∥v_{0}{∥}_{V^{3}}^{2}+\frac{\varkappa }{2}{∥v_{0}∥}_{V^{1}}^{2};\\ \frac{\varkappa }{2}{∥v\left(\tau \right)∥}_{V^{1}}^{2}⩽\frac{T}{\varkappa }{∥f∥}_{L_{2}\left(0,T;V^{-1}\right)}^{2}+\frac{\varkappa }{4}{∥v∥}_{C(\left[0,T\right],V^{1})}^{2}+\frac{1}{2}∥v_{0}{∥}_{V^{0}}^{2}+\frac{\varepsilon }{2}∥v_{0}{∥}_{V^{3}}^{2}+\frac{\varkappa }{2}{∥v_{0}∥}_{V^{1}}^{2}. \end{array}$$

The right-hand side of the last two inequalities does not depend on τ. Therefore, we can take the maximum with respect to τ∈[0,T] on the left-hand side:

$$\begin{array}{c} \frac{\varepsilon }{2}{∥v∥}_{C(\left[0,T\right],V^{3})}^{2}⩽\frac{T}{\varkappa }{∥f∥}_{L_{2}\left(0,T;V^{-1}\right)}^{2}+\frac{\varkappa }{4}{∥v∥}_{C(\left[0,T\right],V^{1})}^{2}+\frac{1}{2}∥v_{0}{∥}_{V^{0}}^{2}+\frac{\varepsilon }{2}∥v_{0}{∥}_{V^{3}}^{2}+\frac{\varkappa }{2}{∥v_{0}∥}_{V^{1}}^{2};\\ \frac{\varkappa }{2}{∥v∥}_{C(\left[0,T\right],V^{1})}^{2}⩽\frac{T}{\varkappa }{∥f∥}_{L_{2}\left(0,T;V^{-1}\right)}^{2}+\frac{\varkappa }{4}{∥v∥}_{C(\left[0,T\right],V^{1})}^{2}+\frac{1}{2}∥v_{0}{∥}_{V^{0}}^{2}+\frac{\varepsilon }{2}∥v_{0}{∥}_{V^{3}}^{2}+\frac{\varkappa }{2}{∥v_{0}∥}_{V^{1}}^{2}. \end{array}$$

This proves (22) and (23). □

**Theorem** **4.** *If $v\in E_{2}$ is a solution of operator Equation (21) for some λ∈[0,1], it satisfies the following estimates:*

(26) $$\varepsilon ∥v^{\prime }{∥}_{L_{2}\left(0,T;V^{3}\right)}⩽C_{23},$$

(27) $$∥v^{\prime }{∥}_{L_{2}\left(0,T;V^{-1}\right)}⩽\frac{2C_{23}}{C_{8}}.$$

*where*

$$C_{23}=C_{24}{∥f∥}_{L_{2}\left(0,T;V^{-1}\right)}+C_{29}\left(\frac{C_{21}+2\varepsilon {∥v_{0}∥}_{V^{3}}^{2}}{\varkappa }\right)+\nu C_{30}\sqrt{\frac{C_{21}+2\varepsilon {∥v_{0}∥}_{V^{3}}^{2}}{\varkappa }}.$$

**Proof.**  Let $v\in E_{2}$ be a solution of the problem (21). Then it satisfies the following operator equation

(28) $$v^{\prime }+\varkappa Av^{\prime }+\varepsilon Dv^{\prime }+\lambda N\left(v\right)-\lambda B_{1}\left(v\right)-\lambda \varkappa B_{2}\left(v\right)-\lambda \varkappa B_{3}\left(v\right)+2\lambda \varkappa B_{4}\left(v\right)=\lambda f.$$

Therefore,

(29) $$\begin{array}{c} {∥v^{\prime }+\varkappa Av^{\prime }+\varepsilon Dv^{\prime }∥}_{L_{2}\left(0,T;V^{-3}\right)}=\\ ={∥\lambda f+\lambda B_{1}\left(v\right)-\lambda N\left(v\right)+\lambda \varkappa B_{2}\left(v\right)+\lambda \varkappa B_{3}\left(v\right)-2\lambda \varkappa B_{4}\left(v\right)∥}_{L_{2}\left(0,T;V^{-3}\right)}. \end{array}$$

The right-hand side of the inequality can be estimated in the following way:

$${∥\lambda f-\lambda N\left(v\right)+\lambda B_{1}\left(v\right)+\lambda \varkappa B_{2}\left(v\right)+\lambda \varkappa B_{3}\left(v\right)-2\lambda \varkappa B_{4}\left(v\right)∥}_{L_{2}\left(0,T;V^{-3}\right)}⩽$$

$$⩽{\lambda ∥f∥}_{L_{2}\left(0,T;V^{-3}\right)}+{\lambda ∥N\left(v\right)∥}_{L_{2}\left(0,T;V^{-3}\right)}+\lambda {∥B_{1}\left(v\right)∥}_{L_{2}\left(0,T;V^{-3}\right)}+$$

$$+\lambda \varkappa ∥B_{2}{\left(v\right)∥}_{L_{2}\left(0,T;V^{-3}\right)}+\lambda \varkappa ∥B_{3}{\left(v\right)∥}_{L_{2}\left(0,T;V^{-3}\right)}+2\lambda \varkappa {∥B_{4}\left(v\right)∥}_{L_{2}\left(0,T;V^{-3}\right)}⩽$$

$$\leq {∥f∥}_{L_{2}\left(0,T;V^{-3}\right)}+{∥N\left(v\right)∥}_{L_{2}\left(0,T;V^{-3}\right)}+{∥B_{1}\left(v\right)∥}_{L_{2}\left(0,T;V^{-3}\right)}+$$

$$+\varkappa ∥B_{2}{\left(v\right)∥}_{L_{2}\left(0,T;V^{-3}\right)}+\varkappa ∥B_{3}{\left(v\right)∥}_{L_{2}\left(0,T;V^{-3}\right)}+2\varkappa {∥B_{4}\left(v\right)∥}_{L_{2}\left(0,T;V^{-3}\right)}⩽$$

using the continuous embedding $L_{2}\left(0,T;V^{-1}\right)\subset L_{2}\left(0,T;V^{-3}\right)$ and estimates (15), (16), (18) and (20) we have

$$⩽C_{24}{∥f∥}_{L_{2}\left(0,T;V^{-1}\right)}+C_{25}{∥v∥}_{C(\left[0,T\right],V^{1})}^{2}+C_{26}{∥v∥}_{C(\left[0,T\right],V^{1})}+$$

$$+2\varkappa C_{27}{∥v∥}_{C(\left[0,T\right],V^{1})}^{2}+C_{28}{∥v∥}_{C(\left[0,T\right],V^{1})}^{2}=$$

$$=C_{24}{∥f∥}_{L_{2}\left(0,T;V^{-1}\right)}+C_{29}{∥v∥}_{C(\left[0,T\right],V^{1})}^{2}+C_{30}{∥v∥}_{C(\left[0,T\right],V^{1})}⩽$$

in view of a priori estimate (23)

$$\begin{array}{c} ⩽C_{24}{∥f∥}_{L_{2}\left(0,T;V^{-1}\right)}+C_{29}\left(\frac{C_{21}+2\varepsilon {∥v_{0}∥}_{V^{3}}^{2}}{\varkappa }\right)+C_{30}\sqrt{\frac{C_{21}+2\varepsilon {∥v_{0}∥}_{V^{3}}^{2}}{\varkappa }}. \end{array}$$

Now using the estimate (13):

$$\begin{array}{c} \varepsilon ∥v^{\prime }{∥}_{L_{2}\left(0,T;V^{3}\right)}⩽{∥v^{\prime }+\varkappa Av^{\prime }+\varepsilon Dv^{\prime }∥}_{L_{2}\left(0,T;V^{-3}\right)} \end{array}$$

on the left-hand side of (29), we get the required inequality (26).

The estimate (27) is obtained in the following way. As above, *v* satisfies the Equation (28), and, therefore,

$$\begin{array}{c} ∥v^{\prime }+\varkappa Av^{\prime }{∥}_{L_{2}\left(0,T;V^{-3}\right)}=\\ =∥-\varepsilon Dv^{\prime }+\lambda f+\lambda B_{1}\left(v\right)-\lambda N\left(v\right)+\lambda \varkappa B_{2}\left(v\right)+\lambda \varkappa B_{3}\left(v\right)-2\lambda \varkappa B_{4}{\left(v\right)∥}_{L_{2}\left(0,T;V^{-3}\right)}⩽\\ ⩽\varepsilon {∥Dv^{\prime }∥}_{L_{2}\left(0,T;V^{-3}\right)}+∥\lambda f+\lambda B_{1}\left(v\right)-\lambda N\left(v\right)+\lambda \varkappa B_{2}\left(v\right)+\lambda \varkappa B_{3}\left(v\right)-\\ -2\lambda \varkappa B_{4}{\left(v\right)∥}_{L_{2}\left(0,T;V^{-3}\right)}⩽\varepsilon {∥v^{\prime }∥}_{L_{2}\left(0,T;V^{3}\right)}+C_{23}⩽2C_{23}. \end{array}$$

Thus, we get the estimate

$$∥v^{\prime }+\varkappa Av^{\prime }{∥}_{L_{2}\left(0,T;V^{-3}\right)}⩽2C_{23}.$$

From this inequality and from estimate (14):

$$C_{8}∥v^{\prime }{∥}_{L_{2}\left(0,T;V^{-1}\right)}⩽{∥\left(J+\varkappa A\right)v^{\prime }∥}_{L_{2}\left(0,T;V^{-3}\right)}$$

(27) follows. □

Theorems 3 and 4 imply the following Theorem:

**Theorem** **5.** *If v is a solution of the operator Equation *(21)* it satisfies the following a priory estimate:*

(30) $$\begin{array}{c} {∥v∥}_{E_{2}}⩽C_{26}=\sqrt{\frac{C_{21}}{\varepsilon }+2{∥v_{0}∥}_{V^{3}}^{2}}+\frac{C_{23}}{\varepsilon }. \end{array}$$

## 5. Existence Theorem for the Auxiliary Problem

**Theorem** **6.** *Let $\Omega \subset R^{n}$, n=2,3, be bounded domain with smooth boundary and $v_{0}\in V^{3}$, $f\in L_{2}\left(0,T;V^{-1}\right)$. Then there is a weak solution $v\in E_{2}$ of the operator Equation *(12)*.*

**Proof.**  The prove of this Theorem is based on the Leray–Schauder topological degree theory for compact vector fields. If we consider the ball $B_{R}\subset E_{2}$ of radius $R=C_{26}+1$ then all solutions of the family of Equation (21) will be in this ball by virtue of a priori estimate (30).

We have the continuous operator $L^{-1}:L_{2}\left(0,T;V^{-3}\right)\times V^{3}\to E_{2}$ and compact mapping

$$\left[-K\left(\cdot \right)+\left(f,v_{0}\right)\right]:E_{2}\to L_{2}\left(0,T;V^{-3}\right)\times V^{3}$$

(see Lemma 9). Thus, the mapping

$$G:\left[0,1\right]\times E_{2}\to E_{2},\;G\left(\lambda ,v\right)=\lambda L^{-1}\left[-K\left(v\right)+\left(f,v_{0}\right)\right]$$

is compact in (λ,v) (jointly in λ and *v*). In other word we have the compact vector field

Φ(λ,v)=v−G(λ,v).

Therefore, the Leray–Schauder topological degree $\deg_{LS}\left(\Phi ,B_{R},0\right)$ is defined. By the homotopy invariance and normalization condition of the degree we get

$$\deg_{LS}\left(\Phi \left(0,\cdot \right),B_{R},0\right)={deg}_{LS}\left(\Phi \left(1,\cdot \right),B_{R},0\right)=1.$$

So why, the equation

$$v-L^{-1}\left[-K\left(v\right)+\left(f,v_{0}\right)\right]=0$$

and, therefore, the Equation (12), and, therefore, the auxiliary problem, have a solution $v\in E_{2}$. □

## 6. Proof of Theorem 1

**Proof.**  Since the space $V^{3}$ is dense in $V^{1},$ there is a sequence ${\left(v_{0}\right)}_{m}\in V^{3}$ converging to ${\left(v_{0}\right)}_{*}\in V^{1}.$ If ${\left(v_{0}\right)}_{*}\equiv 0,$ then put

$${\left(v_{0}\right)}_{m}\equiv 0,\;\varepsilon_{m}=\frac{1}{m}.$$

But if $∥{\left(v_{0}\right)}_{*}{∥}_{V^{1}}\neq 0,$ then $∥{\left(v_{0}\right)}_{m}{∥}_{V^{3}}\neq 0$ if *m* is sufficiently large. Then we set $\varepsilon_{m}=\frac{1}{m∥{\left(v_{0}\right)}_{m}{∥}_{V^{3}}^{2}}.$

Then the sequence $\left\{\varepsilon_{m}\right\}$ converges to zero as m→+∞. The following estimate holds:

(31) $$\varepsilon_{m}{∥{\left(v_{0}\right)}_{m}∥}_{V^{3}}^{2}⩽1.$$

By Theorem 6 for any $\varepsilon_{m}$ and ${\left(v_{0}\right)}_{m}$ there exists a weak solution $v_{m}\in E_{2}\subset E_{1}$ of the approximation problem. Thus, each $v_{m}$ satisfies the equation

(32) $$\begin{array}{c} \int_{\Omega }v_{m}^{\prime }\varphi \;dx-\int_{\Omega }\sum_{i,j=1}^{n}{\left(v_{m}\right)}_{i}{\left(v_{m}\right)}_{j}\frac{\partial \varphi_{j}}{\partial x_{i}}\;dx+2\int_{\Omega }\mu (I_{2}\left(v_{m}\right)E\left(v_{m}\right):E\left(\varphi \right)\;dx+\\ +\varepsilon_{m}\int_{\Omega }\nabla \left(\Delta v_{m}^{\prime }\right):\nabla \left(\Delta \varphi \right)dx+\varkappa \int_{\Omega }\nabla v_{m}^{\prime }:\nabla \varphi \;dx-\\ -\varkappa \int_{\Omega }\sum_{i,j,k=1}^{n}{\left(v_{m}\right)}_{k}\frac{\partial {\left(v_{m}\right)}_{i}}{\partial x_{j}}\frac{\partial^{2}\varphi_{j}}{\partial x_{i}\partial x_{k}}\;dx-\varkappa \int_{\Omega }\sum_{i,j,k=1}^{n}{\left(v_{m}\right)}_{k}\frac{\partial {\left(v_{m}\right)}_{j}}{\partial x_{i}}\frac{\partial^{2}\varphi_{j}}{\partial x_{i}\partial x_{k}}\;dx+\\ +2\varkappa \int_{\Omega }\left(E\left(v_{m}\right)W_{\rho }\left(v_{m}\right)-W_{\rho }\left(v_{m}\right)E\left(v_{m}\right)\right):\nabla \varphi \;dx=\langle f,\varphi \rangle . \end{array}$$

and the initial condition

(33) $$\begin{array}{c} v_{m}{|}_{t=0}\left(x\right)={\left(v_{0}\right)}_{m}\left(x\right),\;x\in \Omega . \end{array}$$

Since the sequence $\left\{{\left(v_{0}\right)}_{m}\right\}$ converges in $V^{1},$ it is bouned in the norm of $V^{1}.$ Therefore,

(34) $$\begin{array}{c} 2∥{\left(v_{0}\right)}_{m}{∥}_{V^{0}}^{2}+2\varkappa {∥{\left(v_{0}\right)}_{m}∥}_{V^{1}}^{2}⩽C_{27}, \end{array}$$

where $C_{27}$ is a constant which doesn’t depend on m.

Recall that the constant $C_{21}$ from the inequality (23) depends on m:

$$C_{21}=\frac{4T}{\varkappa }{∥f∥}_{L_{2}\left(0,T;V^{-1}\right)}^{2}+2∥{\left(v_{0}\right)}_{m}{∥}_{V^{0}}^{2}+2\varkappa {∥{\left(v_{0}\right)}_{m}∥}_{V^{1}}^{2}.$$

Hence, with the help of (34), it can be estimated in the following way:

(35) $$C_{21}⩽\frac{4T}{\varkappa }{∥f∥}_{L_{2}\left(0,T;V^{-1}\right)}^{2}+C_{27}=C_{28}.$$

Thus, in view of inequalities (31) and (35) from (23) we get that

(36) $$\varkappa ∥v_{m}{∥}_{C(\left[0,T\right],V^{1})}^{2}⩽C_{28}+2.$$

Similarly from (31) and (35) with the help of inequalities (26) and (27) we get:

(37) $$\varepsilon ∥v_{m}^{\prime }{∥}_{L_{2}\left(0,T;V^{3}\right)}⩽C_{24}{∥f∥}_{L_{2}\left(0,T;V^{-1}\right)}+C_{29}\left(\frac{C_{28}+2}{\varkappa }\right)+C_{30}\sqrt{\frac{C_{28}+2}{\varkappa }},$$

(38) $$∥v_{m}^{\prime }{∥}_{L_{2}\left(0,T;V^{-1}\right)}⩽\frac{2C_{24}}{C_{8}}{∥f∥}_{L_{2}\left(0,T;V^{-1}\right)}+2C_{29}\left(\frac{C_{28}+2}{\varkappa C_{8}}\right)+\frac{2C_{30}}{C_{8}}\sqrt{\frac{C_{28}+2}{\varkappa }}.$$

Since the embeddings

$$C\left(\left[0,T\right],V^{1}\right)\subset L_{2}\left(0,T;V^{1}\right)\;\;\mathrm{and}\;\;C\left(\left[0,T\right],V^{1}\right)\subset L_{\infty }\left(0,T;V^{1}\right)$$

are continuous, it follows from (36) and (38) that without loss of generality (passing to a subsequence if needed) we have

$$\begin{array}{c} v_{m}⇀v_{*}\;\mathrm{weakly}\;\mathrm{in}\;\;L_{2}\left(0,T;V^{1}\right)\;\mathrm{as}\;\;m\to +\infty ;\\ v_{m}⇀v_{*}\;*-\mathrm{weakly}\;\mathrm{in}\;\;L_{\infty }\left(0,T;V^{1}\right)\;\mathrm{as}\;\;m\to +\infty ;\\ v_{m}^{\prime }⇀v_{*}^{\prime }\;\mathrm{weakly}\;\mathrm{in}\;\;L_{2}\left(0,T;V^{-1}\right)\;\mathrm{as}\;\;m\to +\infty . \end{array}$$

Then by definition of weak convergence

$$\begin{array}{c} \int_{\Omega }\mu \left(I_{2}\left(v_{m}\right)\right)E\left(v_{m}\right):E\left(\varphi \right)\;dx\to \int_{\Omega }\mu \left(I_{2}\left(v_{*}\right)\right)E\left(v_{*}\right):E\left(\varphi \right)\;dx\;\mathrm{as}\;m\to +\infty ,\;\;\varphi \in V^{3};\\ \int_{\Omega }v_{m}^{\prime }\varphi \;dx+\varkappa \int_{\Omega }\nabla v_{m}^{\prime }:\nabla \varphi \;dx=\langle \left(J+\varkappa A\right)v_{m}^{\prime },\varphi \rangle \to \langle \left(J+\varkappa A\right)v_{*}^{\prime },\varphi \rangle . \end{array}$$

as m→+∞.

In view of the estimate (37) we have that $\varepsilon_{m}v_{m}^{\prime }⇀u$ converges weakly in $L_{2}\left(0,T;V^{3}\right)$. On the other hand, for any $\chi \in D\left(\left[0,T\right]\right),\varphi \in V^{5}$ we have

$$\begin{array}{c} \lim_{m\to \infty }\left|\varepsilon_{m}\int_{0}^{T}\int_{\Omega }\nabla \left(\Delta v_{m}^{\prime }\left(t\right)\right):\nabla \left(\Delta \varphi \right)\;dx\chi \left(t\right)dt\right|=\lim_{m\to \infty }\varepsilon_{m}\left|\int_{0}^{T}\int_{\Omega }\Delta v_{m}^{\prime }\left(t\right)\Delta^{2}\varphi \;dx\chi \left(t\right)dt\right|=\\ =\lim_{m\to \infty }\varepsilon_{m}\left|\int_{0}^{T}\int_{\Omega }\nabla \left(v_{m}^{\prime }\left(t\right)\right):\nabla \left(\Delta^{2}\varphi \right)\;dx\chi \left(t\right)dt\right|=\\ =\lim_{m\to \infty }\varepsilon_{m}\lim_{m\to \infty }\left|\int_{0}^{T}\int_{\Omega }\nabla \left(v_{m}^{\prime }\left(t\right)\right):\nabla \left(\Delta^{2}\varphi \right)\;dx\chi \left(t\right)dt\right|=\\ =\lim_{m\to \infty }\varepsilon_{m}\lim_{m\to \infty }\left|\int_{\Omega }\left(\int_{0}^{T}\nabla \left(v_{m}^{\prime }\left(t\right)\right)\chi \left(t\right)dt\right):\nabla \left(\Delta^{2}\varphi \right)\;dx\right|=\\ =\lim_{m\to \infty }\varepsilon_{m}\lim_{m\to \infty }\left|\int_{\Omega }\left(\int_{0}^{T}\nabla v_{m}\left(t\right)\frac{\partial \chi (t)}{\partial t}dt\right):\nabla \left(\Delta^{2}\varphi \right)\;dx\right|=\\ =\lim_{m\to \infty }\varepsilon_{m}\lim_{m\to \infty }\left|\int_{0}^{T}\int_{\Omega }\nabla v_{m}\left(t\right):\nabla \left(\Delta^{2}\varphi \right)\;dx\frac{\partial \chi (t)}{\partial t}dt\right|= \end{array}$$

Since $v_{m}$ weakly converges to $v_{*}$ in $L_{2}\left(0,T;V^{1}\right)$ (and, therefore, converges to $v_{*}$ in the sense of distributions) the latter expression equals

$$\begin{array}{c} =\left|\int_{0}^{T}\int_{\Omega }\nabla v_{*}\left(t\right):\nabla \left(\Delta^{2}\varphi \right)\;dx\frac{\partial \chi (t)}{\partial t}dt\right|\lim_{m\to \infty }\varepsilon_{m}=0. \end{array}$$

Thus, by uniqueness of the weak limit we get

$$\varepsilon_{m}\int_{\Omega }\nabla \left(\Delta v_{m}^{\prime }\right):\nabla \left(\Delta \varphi \right)\;dx\to 0\;\mathrm{as}\;\;m\to +\infty .$$

Using Theorem 2 we have the compact embedding

$$F=\left\{v:v\in C\left(\left[0,T\right],V^{1}\right);v^{\prime }\in L_{2}\left(0,T;V^{-1}\right)\right\}\subset C\left(\left[0,T\right],L_{4}{\left(\Omega \right)}^{n}\right).$$

Hence, taking into account estimates (36) and (38), we obtain

(39) $$\begin{array}{c} v_{m}\to v_{*},\;\mathrm{strongly}\;\mathrm{in}\;\;C\left(\left[0,T\right],L_{4}{\left(\Omega \right)}^{n}\right)\;\mathrm{as}\;\;m\to +\infty . \end{array}$$

Thus, we get

$$\begin{array}{c} \int_{\Omega }\sum_{i,j=1}^{n}{\left(v_{m}\right)}_{i}{\left(v_{m}\right)}_{j}\frac{\partial \varphi_{j}}{\partial x_{i}}\;dx\to \int_{\Omega }\sum_{i,j=1}^{n}{\left(v_{*}\right)}_{i}{\left(v_{*}\right)}_{j}\frac{\partial \varphi_{j}}{\partial x_{i}}\;dx\;\mathrm{as}\;\;m\to +\infty . \end{array}$$

For the remaining integrals we have

$$\int_{\Omega }\sum_{i,j,k=1}^{n}{\left(v_{m}\right)}_{k}\frac{\partial {\left(v_{m}\right)}_{i}}{\partial x_{j}}\frac{\partial^{2}\varphi_{j}}{\partial x_{i}\partial x_{k}}\;dx\to \int_{\Omega }\sum_{i,j,k=1}^{n}{\left(v_{*}\right)}_{k}\frac{\partial {\left(v_{*}\right)}_{i}}{\partial x_{j}}\frac{\partial^{2}\varphi_{j}}{\partial x_{i}\partial x_{k}}\;dx;$$

$$\int_{\Omega }\sum_{i,j,k=1}^{n}{\left(v_{m}\right)}_{k}\frac{\partial {\left(v_{m}\right)}_{j}}{\partial x_{i}}\frac{\partial^{2}\varphi_{j}}{\partial x_{i}\partial x_{k}}\;dx\to \int_{\Omega }\sum_{i,j,k=1}^{n}{\left(v_{*}\right)}_{k}\frac{\partial {\left(v_{*}\right)}_{j}}{\partial x_{i}}\frac{\partial^{2}\varphi_{j}}{\partial x_{i}\partial x_{k}}\;dx,$$

as m→+∞. Indeed, here the sequence $v_{m}$ converges to $v_{*}$ strongly in $C(\left[0,T\right],L_{4}\left(\Omega \right))$ and $\nabla (v_{m})$ converges to $\nabla v_{*}$ weakly in $L_{4}\left(0,T;L_{2}\left(\Omega \right)\right).$ Thus, their product converges to the product of their limits.

In the last term we have

$$|\int_{\Omega }\left(E\left(v_{m}\right)W_{\rho }\left(v_{m}\right)-E\left(v_{*}\right)W_{\rho }\left(v_{*}\right)\right):\nabla \varphi \;dx|=$$

$$=|\int_{\Omega }\left(E\left(v_{m}\right)\left(W_{\rho }\left(v_{m}\right)-W_{\rho }\left(v_{*}\right)\right)+\left(E\left(v_{m}\right)-E\left(v_{*}\right)\right)W_{\rho }\left(v_{*}\right)\right):\nabla \varphi \;dx|\leq$$

$$\leq ∥E\left(v_{m}\right){∥}_{L_{2}{\left(\Omega \right)}^{n^{2}}}{∥\nabla \varphi ∥}_{L_{2}{\left(\Omega \right)}^{n^{2}}}{∥W_{\rho }\left(v_{m}-v_{*}\right)∥}_{L_{\infty }{\left(\Omega \right)}^{n^{2}}}+$$

$$+∥W_{\rho }\left(v_{*}\right){∥}_{L_{\infty }{\left(\Omega \right)}^{n^{2}}}|\int_{\Omega }E\left(v_{m}-v_{*}\right):\nabla \varphi \;dx|\leq$$

$$\leq C_{31}(∥E\left(v_{m}\right){∥}_{L_{2}{\left(\Omega \right)}^{n^{2}}}{∥\nabla \varphi ∥}_{L_{2}{\left(\Omega \right)}^{n^{2}}}{∥v_{m}-v_{*}∥}_{L_{2}{\left(\Omega \right)}^{n}}+$$

$$+∥W_{\rho }\left(v_{*}\right){∥}_{L_{\infty }{\left(\Omega \right)}^{n^{2}}}|\int_{\Omega }E\left(v_{m}-v_{*}\right):\nabla \varphi \;dx|)\leq$$

$$\leq C_{32}(∥E\left(v_{m}\right){∥}_{L_{2}{\left(\Omega \right)}^{n^{2}}}{∥\nabla \varphi ∥}_{L_{2}{\left(\Omega \right)}^{n^{2}}}{∥v_{m}-v_{*}∥}_{L_{4}{\left(\Omega \right)}^{n}}+$$

$$+∥W_{\rho }\left(v_{*}\right){∥}_{L_{\infty }{\left(\Omega \right)}^{n^{2}}}|\int_{\Omega }E\left(v_{m}-v_{*}\right):\nabla \varphi \;dx|).$$

As above we get that $v_{m}\to v_{*}$ strongly in $C(\left[0,T\right],L_{4}\left(\Omega \right))$ and $\nabla \left(v_{m}\right)⇀\nabla v_{*}$ weakly in $L_{4}\left(0,T;L_{2}\left(\Omega \right)\right),$ we obtain

$$\begin{array}{c} \int_{\Omega }E\left(v_{m}\right)W_{\rho }\left(v_{m}\right):\nabla \varphi \;dx\to \int_{\Omega }E\left(v_{*}\right)W_{\rho }\left(v_{*}\right):\nabla \varphi \;dx\;\mathrm{as}\;\;m\to +\infty .\\ \int_{\Omega }W_{\rho }\left(v_{m}\right)E\left(v_{m}\right):\nabla \varphi \;dx\to \int_{\Omega }W_{\rho }\left(v_{*}\right)E\left(v_{*}\right):\nabla \varphi \;dx\;\mathrm{as}\;\;m\to +\infty . \end{array}$$

Pass to limit in (32) as m→+∞. We get a function $v_{*}$ satisfying

$$\begin{array}{c} \langle \left(J+\varkappa A\right)\frac{\partial v_{*}}{\partial t},\varphi \rangle -\int_{\Omega }\sum_{i,j=1}^{n}{\left(v_{*}\right)}_{i}{\left(v_{*}\right)}_{j}\frac{\partial \varphi_{j}}{\partial x_{i}}\;dx+2\int_{\Omega }\mu \left(I_{2}\left(v_{*}\right)\right)E\left(v_{*}\right):E\left(\varphi \right)\;dx-\\ -\varkappa \int_{\Omega }\sum_{i,j,k=1}^{n}{\left(v_{*}\right)}_{k}\frac{\partial {\left(v_{*}\right)}_{i}}{\partial x_{j}}\frac{\partial^{2}\varphi_{j}}{\partial x_{i}\partial x_{k}}\;dx-\varkappa \int_{\Omega }\sum_{i,j,k=1}^{n}{\left(v_{*}\right)}_{k}\frac{\partial {\left(v_{*}\right)}_{j}}{\partial x_{i}}\frac{\partial^{2}\varphi_{j}}{\partial x_{i}\partial x_{k}}\;dx+\\ +2\varkappa \int_{\Omega }\left(E\left(v_{*}\right)W_{\rho }\left(v_{*}\right)-W_{\rho }\left(v_{*}\right)E\left(v_{*}\right)\right):\nabla \varphi \;dx=\langle f,\varphi \rangle . \end{array}$$

As we have a strong convergence (39) then we get that this obtained function $v_{*}$ satisfies the initial condition $v_{*}{|}_{t=0}={\left(v_{0}\right)}_{*}$. So, we prove Theorem 1. □

## 7. Optimal Feedback Control Problem

In this section based on the topological approximation approach to mathematical hydrodynamics problems we prove the existence of an optimal feedback control for the (6)–(9) problem. First, we formulate the notion of a solution to the problem under consideration and the main result of this section.

Consider the multi-valued mapping $\Psi :E_{1}⊸L_{2}\left(0,T;V^{-1}\right)$ as a control function. We will assume that Ψ satisfies the following conditions:

(Ψ1) The mapping Ψ is defined on the space $E_{1}$ and has non-empty, compact, convex values;
(Ψ2) The mapping Ψ is upper semicontinuous and compact;
(Ψ3) The mapping Ψ is globally bounded, that is, there exists a constant M>0 such that
  $${∥\Psi \left(v\right)∥}_{L_{2}\left(0,T;V^{-1}\right)}:=\sup\left\{{∥u∥}_{L_{2}\left(0,T;V^{-1}\right)}:u\in \Psi \left(v\right)\right\}\leq M\;\mathrm{for}\;\mathrm{all}\;v\in E_{1};$$
(Ψ4) Ψ is weakly closed in the following sense: if ${\left\{v_{l}\right\}}_{l=1}^{\infty }\subset E_{1},v_{l}⇀v_{0}\;,u_{l}\in \Psi \left(v_{l}\right)$ and $u_{l}\to u_{0}\;\;$ in $L_{2}\left(0,T;V^{-1}\right)$ then $u_{0}\in \Psi \left(v_{0}\right)$.

For completeness, we give an example of such a multi-valued mapping. Let continuous mappings $f_{i}:E_{1}\to L_{2}\left(0,T;V^{-1}\right),\;i=1,2,\ldots m$ satisfy the following conditions:

1. $f_{i}$ is globally bounded and makes a bounded set relatively compact;
2. $f_{i}$—weakly closed, i.e., ${\left\{v_{l}\right\}}_{l=1}^{\infty }\subset E_{1},v_{l}⇀v_{0},\;f_{i}\left(v_{l}\right)\to u_{0}$ follows $u_{0}=f_{i}\left(v_{0}\right).$

We define a multimap with feedback $U:E_{1}\to L_{2}\left(0,T;V^{-1}\right)$ as:

$$U\left(v\right)=\{u=\sum_{i=1}^{m}\lambda_{i}f_{i}\left(v\right)\;:\;\sum_{i=1}^{m}\lambda_{i}=1\}.$$

It is easy to see that *U* satisfies all the conditions of the multi-valued mapping Ψ.

We will consider a weak formulation of the optimal feedback control problem for the initial–boundary value problem (6)–(9). By feedback, we mean the following condition:

(40) f∈Ψ(v).

We will assume that the initial condition $v_{0}$ belongs to the space $V^{1}.$

**Definition** **2.** *A pair of functions $\left(v,f\right)\in E_{1}\times L_{2}\left(0,T;V^{-1}\right)$ is called a weak solution to the feedback control problem *(6)*–*(9)*, *(40)* if it is for any $\varphi \in V^{3}$ and almost all t∈(0,T) satisfies the feedback condition (40), the identity *

(41) $$\begin{array}{c} \frac{d}{dt}\int_{\Omega }v\varphi \;dx-\int_{\Omega }\sum_{i,j=1}^{n}v_{i}v_{j}\frac{\partial \varphi_{j}}{\partial x_{i}}\;dx+2\int_{\Omega }\mu \left(I_{2}\left(v\right)\right)E\left(v\right):E\left(\varphi \right)\;dx+\varkappa \frac{d}{dt}\int_{\Omega }\nabla v:\nabla \varphi \;dx-\\ -\varkappa \int_{\Omega }\sum_{i,j,k=1}^{n}v_{k}\frac{\partial v_{i}}{\partial x_{j}}\frac{\partial^{2}\varphi_{j}}{\partial x_{i}\partial x_{k}}\;dx-\varkappa \int_{\Omega }\sum_{i,j,k=1}^{n}v_{k}\frac{\partial v_{j}}{\partial x_{i}}\frac{\partial^{2}\varphi_{j}}{\partial x_{i}\partial x_{k}}\;dx+\\ +2\varkappa \int_{\Omega }\left(E\left(v\right)W_{\rho }\left(v\right)-W_{\rho }\left(v\right)E\left(v\right)\right):\nabla \varphi \;dx=\langle f,\varphi \rangle \end{array}$$

*as well as the initial condition*

(42) $$v\left(0\right)=v_{0}.$$

The first result of this section is the following Theorem:

**Theorem** **7.** *Let the mapping Ψ satisfy the conditions (Ψ1)–(Ψ4). Then there exists at least one weak solution to the feedback control problem *(6)–(9)*, *(40)*.*

Denote by $\Sigma \subset E_{1}\times L_{2}\left(0,T;V^{-1}\right)$ the set of all weak solutions of the problem (6)–(9), (40). Consider an arbitrary functional Φ:Σ→R, satisfying the following conditions:

(Φ1) There exists a number γ such that Φ(v,f)⩾γ for all (v,f)∈Σ.
(Φ2) If $v_{m}⇀v_{*}$ in $E_{1}$ and $f_{m}\to f_{*}$ in $L_{2}\left(0,T;V^{-1}\right),$ then $\Phi \left(v_{*},f_{*}\right)⩽\underset{m\to \infty }{\underline{\lim}}\Phi \left(v_{m},f_{m}\right).$

As an example of such a quality functional, consider:

$$\begin{array}{c} \Phi \left(v,f\right)=\int_{0}^{T}{(∥v\left(t,\cdot \right)-U\left(t,\cdot \right)∥}_{V^{1}}^{2}{+∥f\left(t,\cdot \right)-F\left(t,\cdot \right)∥}_{V^{-1}}^{2})dt. \end{array}$$

Here *U* and *F* are given speed and external force.

The main result of this section is the following Theorem.

**Theorem** **8.** If the mapping Ψ satisfies the conditions (Ψ1)–(Ψ4) and the functional Φ satisfies the conditions (Φ1)–(Φ2), then the problem of optimal control with feedback *(6)–(9)*, *(40)* has at least one weak solution $\left(v_{*},f_{*}\right)$ such that $\Phi \left(v_{*},f_{*}\right)=\underset{(v,f)\in \Sigma }{\inf}\Phi \left(v,f\right).$

To prove these Theorems we at first consider the auxiliary problem with some small parameter ε>0: We need to find a pair of functions $\left(v,f\right)\in E_{2}\times L_{2}\left(0,T;V^{-1}\right),$ satisfying for any $\varphi \in V^{3}$ and almost all t∈(0,T) the feedback condition (40), identity

$$\int_{\Omega }\frac{\partial v}{\partial t}\varphi \;dx-\int_{\Omega }\sum_{i,j=1}^{n}v_{i}v_{j}\frac{\partial \varphi_{j}}{\partial x_{i}}\;dx+2\int_{\Omega }\mu \left(I_{2}\left(v\right)\right)E\left(v\right):E\left(\varphi \right)\;dx+\varkappa \int_{\Omega }\nabla \left(\frac{\partial v}{\partial t}\right):\nabla \varphi \;dx+$$

(43) $$\begin{array}{c} +\varepsilon \int_{\Omega }\nabla \left(\Delta (\frac{\partial v}{\partial t})\right):\nabla \left(\Delta \varphi \right)\;dx-\varkappa \int_{\Omega }\sum_{i,j,k=1}^{n}v_{k}\frac{\partial v_{i}}{\partial x_{j}}\frac{\partial^{2}\varphi_{j}}{\partial x_{i}\partial x_{k}}\;dx-\\ --\varkappa \int_{\Omega }\sum_{i,j,k=1}^{n}v_{k}\frac{\partial v_{j}}{\partial x_{i}}\frac{\partial^{2}\varphi_{j}}{\partial x_{i}\partial x_{k}}\;dx+2\varkappa \int_{\Omega }\left(E\left(v\right)W_{\rho }\left(v\right)-W_{\rho }\left(v\right)E\left(v\right)\right):\nabla \varphi \;dx=\langle f,\varphi \rangle \end{array}$$

and the initial condition

(44) $$v\left(0\right)=v_{0}.$$

Using the operator treatment (12) we can reformulate our auxiliary problem in the operator form. Thus, the problem of the existence of a feedback control for the approximation problem is equivalent to the problem of the existence of a solution $v\in E_{2}$ satisfying the initial condition (44) of the following operator inclusion:

$$L\left(v\right)+K\left(v\right)\in (\Psi \left(v\right),v_{0}).$$

Let’s introduce the following operator: $Y:E_{1}\to L_{2}\left(0,T;V^{-1}\right)\times V^{3}$; $Y\left(v\right)=(\Psi \left(v\right),v_{0}).$ Then the problem of the existence of a solution $\left(v,f\right)\in E_{2}\times L_{2}\left(0,T;V^{-1}\right)$ of the approximation problem is equivalent to the problem of the existence of a solution $v\in E_{2}$ for next inclusion

(45) $$v\in M\left(v\right),\;\mathrm{where}\;M\left(v\right)=L^{-1}\left(Y\left(v\right)-K\left(v\right)\right).$$

Since the operator $L^{-1}$ is linear and continuous, and the operator *K* is compact, using the conditions (Ψ1)–(Ψ2) we obtain that the multi-valued mapping $M:E_{2}⊸E_{2}$ is compact and has non-empty, convex and compact values.

Consider also the following family of inclusions

(46) v∈λM(v),

where 0≤λ≤1.

**Remark** **1.** *Note that the left side of the operator inclusion *(46)* is exactly the same as the left side of *(21)*. Therefore, the following Theorem hold for operator inclusion:*

**Theorem** **9.** *If v is a solution *(46)* for some λ∈[0,1], then the following estimate holds for it:*

(47) $$\begin{array}{c} {∥v∥}_{E_{2}}⩽C_{26}=\sqrt{\frac{C_{21}}{\varepsilon }+2{∥v_{0}∥}_{V^{3}}^{2}}+\frac{C_{23}}{\varepsilon }. \end{array}$$

**Theorem** **10.** *The operator inclusion (45) has at least one solution $v\in E_{2}.$*

**Proof.**  To prove this Theorem we use topological degree theory for multivalued vector fields (see, for example, [32]). By virtue of the a priori estimate (47), all solutions of the family of operator inclusions (46) lie in the ball $B_{R}\subset E_{2}$ of radius $R=C_{26}+1$ centered at zero. Hence v∉λM(v) for all $\left(v,\lambda \right)\in \partial B_{R}\times \left[0,1\right]$. Using the degree homotopy invariance property and the degree normalization property, we obtain

$$deg\left(I-M,{\bar{B}}_{R},0\right)=deg\left(I,{\bar{B}}_{R},0\right)=1.$$

Since this degree is nonzero, there exists at least one solution $v\in E_{2}$ of the operator inclusion (45). □

Since there exists a solution $v\in E_{2}$ of the inclusion (45), it follows from the above reasoning that the approximation problem has at least one solution $v\in E_{2}.$

Using the results of Theorem 10, we completely repeat the proof of Theorem 1 with a small change related with the right-hand side. Taking into account the a priori estimates (36), (38) and the conditions (Ψ1)−(Ψ4), we can assume without loss of generality that there exists $f_{*}\in L_{2}\left(0,T;V^{-1}\right)$ such that $f_{m}\to f_{*}\in \Psi \left(v_{*}\right)$ as m→∞. From this we obtain that there exists $v_{*}\in E_{1}$ and $f_{*}\in L_{2}\left(0,T;V^{-1}\right)$ satisfying (40), (2) and (42) which completes the proof of the Theorem 7.

From the Theorem 7 we get that the solution set Σ is not empty. Therefore, there exists a minimizing sequence $(v_{l},f_{l})\in \Sigma$ such that

$$\lim_{l\to \infty }\Phi \left(v_{l},f_{l}\right)=\underset{(v,f,)\in \Sigma }{\inf}\Phi \left(v,f\right).$$

As before, using the estimate (47), without loss of generality and passing to a subsequence if necessary, we can assume that $v_{l}⇀v_{*}$ *-weakly in $L_{\infty }\left(0,T;V^{1}\right)$; $v_{l}\to v_{*}$ is strong in $L_{2}\left(0,T;L_{4}\left(\Omega \right)\right)$; $v_{l}⇀v_{*}$ is weak in $L_{2}\left(0,T;V^{-1}\right)$; $f_{l}\to f_{*}\in \Psi \left(v^{*}\right)$ is strong in $L_{2}\left(0,T;V^{-1}\right)$ for m→+∞.

Whence, just as in the previous proof, we get $\;N\left(v_{l}\right)⇀N\left(v_{*}\right)$ weakly in $L_{2}\left(0,T;V^{-1}\right)$; $\left(I+\varkappa A\right)v_{l}^{\prime }⇀\left(I+\varkappa A\right)v_{*}^{\prime }$ weakly in $L_{2}\left(0,T;V^{-3}\right)$; $B_{1}\left(v_{l}\right)\to B_{1}\left(v_{*}\right)$ strongly in $L_{2}\left(0,T;V^{-1}\right)$; $B_{2}\left(v_{l}\right)⇀B_{2}\left(v_{*}\right)$ weakly in $L_{2}\left(0,T;V^{-3}\right)$; $B_{3}\left(v_{l}\right)⇀B_{3}\left(v_{*}\right)$ weakly in $L_{2}\left(0,T;V^{-3}\right)$; $B_{4}\left(v_{l}\right)⇀B_{4}\left(v_{*}\right)$ weakly in $L_{2}\left(0,T;V^{-3}\right)$ for m→+∞.

Passing to the limit in the relation

$$\begin{array}{c} \left(J+\varkappa A\right)v_{l}^{\prime }+N\left(v_{l}\right)-B_{1}\left(v_{l}\right)+\varepsilon Nv_{l}^{\prime }-\varkappa B_{2}\left(v_{l}\right)-\varkappa B_{3}\left(v_{l}\right)+2\varkappa B_{4}\left(v_{l}\right)=f_{l}\in \Psi \left(v_{l}\right), \end{array}$$

we get that $(v^{*},f^{*})\in \Sigma$. Since the functional Φ is lower semicontinuous with respect to the weak topology, we have

$$\Phi \left(v^{*},f^{*}\right)\leq \underset{(v,f)\in \Sigma }{\inf}\Phi \left(v,f\right),$$

which proves that $\left(v^{*},f^{*}\right)$ is the required solution. This completes the proof of Theorem 8.

## 8. Conclusions

To summarize all calculations and proofs in this paper the mathematical model describing the motion of weakly concentrated water polymer solutions is investigated. This model contained the objective Jaumann derivative in the reological relation. Also this model is considered in the case on non-linear viscosity.

The main result of this paper is the solutions existence to initial–boundary value problem and to the feedback control problem for the mathematical model under consideration. Also the existence of an optimal solution to the problem under consideration that gives a minimum to a given bounded quality functional is proved. Results of this paper provide an opportunity for the future investigation of this model. Author proposes the following future research directions for the model under consideration—(1) the numerical analysis of the obtained solutions; (2) the consideration of a turbulence case of this problem; (3) the investigation of alpha-models for this problem and so forth.

## Data Availability

Not applicable.
